# Gut Microbiota-Elicited Aberrant Phosphorylation Induces Protein Structural Anomalies: A Non-Negligible Pathogenic Driver of Autism Spectrum Disorder

**DOI:** 10.3390/microorganisms14091925

**Published:** 2026-09-01

**Authors:** Yongsheng Ge, Zhi Li, Caiyun Yu, Weitong Guo, Guangying Fan, Guiyu Lin, Han Yu, Ying Wang

**Affiliations:** 1 Pediatric Research Institute, Children’s Hospital Affiliated to Shandong University (Jinan Children’s Hospital), Jinan 250022, China; geyongsheng123@foxmail.com (Y.G.); lizhi2019cn@163.com (Z.L.); 15194190560@163.com (C.Y.); 19862137259@163.com (G.F.); 15053177052@163.com (G.L.); yuhan@etyy.com (H.Y.); 2 Jinan Institute of Child Health Care, Children’s Hospital Affiliated to Shandong University (Jinan Children’s Hospital), Jinan 250022, China; etyygwt@163.com

**Keywords:** autism spectrum disorder, gut microbiota, AlphaFold3, molecular docking, phosphoproteomic profiling, fecal microbiota transplantation, gut-brain axis

## Abstract

Autism spectrum disorder (ASD) is a heterogeneous neurodevelopmental condition characterized by impaired social interaction and repetitive stereotyped behaviors, with pathogenic mechanisms that remain incompletely understood. The gut microbiota has emerged as a key regulator of ASD; however, its impact on hippocampal proteomic and phosphoproteomic signatures has not been fully characterized. In this study, we performed fecal microbiota transplantation (FMT) by transferring fecal samples from children with ASD and typically developing controls into antibiotic-treated mice. Gut microbiota from children with ASD induced several ASD-like behaviors in recipient mice, accompanied by aberrant activation of microglia, astrocytes, and neurons, as well as impaired neurogenesis. Phosphoproteomic profiling combined with Gene Ontology (GO) and Kyoto Encyclopedia of Genes and Genomes (KEGG) analyses revealed that differentially phosphorylated proteins were predominantly enriched in synapse-related pathways. ASD-derived microbiota markedly reduced synaptic density, downregulated the synaptic proteins SYP and PSD-95, and inhibited the expression of blood–brain barrier (BBB) tight junction proteins. In silico structural simulations using AlphaFold3 (AF3) and HADDOCK further supported that ASD-FMT may promote abnormal phosphorylation, potentially remodeling SHANK3 and SRRM2 conformations and weakening the binding affinity of SHANK3. Integrative proteomic and phosphoproteomic screening identified FNDC3A as a potential susceptibility-associated protein upregulated by gut microbiota from children with ASD, which was verified in mouse hippocampal tissues and plasma samples from children with ASD using Western blotting and ELISA, respectively. Mechanistically, ASD pathogenesis may be attributable not only to the dysregulation of classical ASD susceptibility genes but also to gut microbiota-driven post-translational phosphorylation remodeling of multiple protein structures. Importantly, this study established an innovative research framework that integrates in silico analyses with wet-lab experiments, yielding novel insights into ASD pathogenesis from the perspective of gut microbiota-induced alterations in the hippocampal phosphoproteome and revealing a plausible molecular mechanism underlying ASD.

## 1. Introduction

Autism spectrum disorder (ASD) represents a heterogeneous group of neurodevelopmental disorders characterized by impaired social communication with repetitive, stereotyped behavioral patterns [1]. Recent epidemiological studies have documented a sharp rise in ASD prevalence, with an estimated incidence of one in 31 children [2]. ASD pathogenesis arises from intricate crosstalk between genetic susceptibility and environmental triggers, and to date, no reliable diagnostic test or curative therapies are available for this disorder [3]. Accumulating evidence indicates that children with ASD exhibit distinct gut microbial profiles compared with those of neurotypical children [4], and this corroborates that the gut–brain axis serves as a key modulator of brain function and behavioral traits [5,6,7]. Gut microbiota dysbiosis may serve as an important factor underlying ASD pathogenesis [8,9].

Despite extensive research efforts, the etiology of ASD has not been fully elucidated. Numerous studies have focused on synaptic pathology underlying ASD, with particular attention to genetic variants, especially duplications and deletions, that regulate synaptic structure and function [10,11]. Such risk genes encompass SHANK, NLGN, NRXN, FMR1, and MECP2, which serve as key mediators of the pathological mechanisms of ASD [3,12]. For instance, SHANK3 is an essential scaffolding protein of the postsynaptic density, and SHANK3 haploinsufficiency has been thoroughly investigated in human ASD patient cohorts and animal models [13,14]. However, overemphasis is placed on protein expression levels, while structural alterations and functional aberrations arising from protein post-translational modifications, especially phosphorylation. As a classic molecular regulatory switch, phosphorylation drives marked conformational alterations within proteins, thereby tightly controlling their functional activity [15,16,17]. Notably, germ-free mice and microbiota-FMT mice receiving gut microbiota from depressed donors displayed global dysregulation of protein phosphorylation [18].

Gut dysbiosis in neurological disorders is commonly accompanied by depleted populations of short-chain fatty acid (SCFA)-producing bacteria, as well as perturbed metabolic profiles of SCFAs, bile acids, tryptophan-derived indoles, trimethylamine N-oxide (TMAO), and lipopolysaccharides [19,20,21,22]. Within the central nervous system, SCFA-triggered phosphorylation cascades confer neuroprotective functions via the modulation of signaling axes including PI3K/Akt and MAPK/ERK, which are involved in neuronal survival, synaptic plasticity, and the regulation of neuroinflammation [23,24,25]. Furthermore, bile acids exert anti-apoptotic effects through survival signals such as the PI3K and mitogen-activated protein kinase pathways [26,27,28,29]. In the model of glutamate-triggered apoptosis in rat cortical neurons, treatment with the bile acid tauroursodeoxycholic acid induces phosphorylation of a pro-apoptotic protein by activating PI3K, thereby suppressing neuronal apoptosis and conferring neuroprotection [26]. Taken together, a variety of metabolites (SCFAs, secondary bile acids, etc.) produced by gut microbiota may directly or indirectly permeate the gut–brain axis or transmit signaling cues along this bidirectional axis, thereby modulating core kinase/phosphatase-mediated signaling cascades in host cells, including the PI3K, MAPK, PKA, and PKC signaling pathways.

To our knowledge, the global protein expression and phosphorylation remodeling in the hippocampus following FMT using gut microbiota from children with ASD remains largely uncharacterized. Therefore, this study aimed to characterize the hippocampal proteomic and phosphoproteomic alterations induced by gut microbiota from children with ASD in a mouse model. We further combined in silico structural variant prediction and molecular docking simulations, followed by a series of comprehensive wet-lab experiments for experimental validation, including behavioral tests, immunofluorescent staining, Western blotting, and Elisa. To our knowledge, this study is the first to dissect the underlying mechanisms by which gut microbiota-derived aberrant phosphorylation resulted in brain disorders in the pathogenesis of ASD. This study also establishes a novel research framework integrating phosphoproteomic profiling with both in silico computational modeling and wet-lab experimentation for research in this field.

## 2. Materials and Methods

### 2.1. Animals

Thirty male C57BL/6 mice at three weeks of age were obtained from Charles River China (Beijing, China) and randomly separated into two groups: the ASD-FMT group (*n* = 15) and Control-FMT group (*n* = 15). All mice were raised in a specific pathogen-free (SPF) facility where ambient temperature was kept at 24 ± 1 °C, relative humidity was maintained between 50% and 70%, and a 12 h light-dark cycle was implemented (light phase: 08:00–20:00). The mice were housed five per cage and supplied with sterilized standard chow and autoclaved tap water available ad libitum. Animals were assigned to behavioral (*n* = 10), omics (*n* = 5), and molecular/histological validation cohorts (10 animals in total). No animals were arbitrarily excluded.

### 2.2. The Sample Processing

Participants enrolled in this study included 20 typically developing healthy children undergoing routine health examinations and 20 gender- and age-matched male children clinically diagnosed with ASD, all recruited from the Children’S Hospital Affiliated with Shandong University. Peripheral blood and fecal specimens were collected from all participants. A subset of 5 fecal samples from each group was randomly selected for bacterial culture to perform subsequent FMT. The diagnosis of ASD was established by experienced pediatric neurologists according to the Diagnostic and Statistical Manual of Mental Disorders (DSM-5) criteria. All participants were male, aged between 3 and 8 years. Ethical approval for the use of blood and fecal samples was granted by the local ethical commission, and written informed consent was signed by the parents or legal guardians prior to sample donation. All clinical evaluations, blood collections, and fecal sample handlings were conducted under strict protocol, and all personal participant data were fully anonymized prior to sample processing and downstream data analysis.

All procedures in this study were approved by the Ethics Committee of the Children’s Hospital Affiliated with Shandong University (Ethical Approval No. SDFE-IRB/P-2023052; Approval Date: 4 September 2023).

Fecal samples derived from individual participants in the children with ASD and healthy control children were subjected to intra-group pooling and homogenization prior to subsequent processing. Samples were resuspended and homogenized in sterile PBS at 5 mL/g, vortexed vigorously to achieve uniform homogeneity, and filtered through sterile gauze to eliminate coarse particulate contaminants. The prepared suspension was centrifuged at 500× *g* for 5 min at 4 °C, and the clarified supernatant was harvested. The supernatant was combined with an equivalent volume of 50% sterile glycerol to establish a final glycerol concentration of 25%. Upon adequate homogenization, the resultant mixture was aliquoted and immediately frozen. All processed specimens were cryopreserved at −80 °C for the FMT procedure.

### 2.3. Antibiotic Treatment and FMT

Prior to FMT, a mixture of broad-spectrum antibiotic pretreatment was performed to deplete indigenous gut microbiota in mice and eliminate their confounding impacts on subsequent FMT outcomes. All experimental mice were intragastrically administered a mixture of broad-spectrum antibiotics comprising vancomycin (50 mg/kg, Aladdin, Shanghai, China), neomycin (100 mg/kg, Aladdin, Shanghai, China), ampicillin (100 mg/kg, Aladdin, Shanghai, China), and metronidazole (100 mg/kg, Aladdin, Shanghai, China) in sterile solvent. The treatment was implemented once daily for five successive days with a gavage volume of 10 mL/kg, consistent with established protocols [30,31]. Notably, the antibiotic working solution was freshly prepared daily to guarantee pharmacological stability and treatment consistency.

Fecal supernatants collected from five ASD donors and five healthy control donors were separately used as a single source for recipient mice. Following antibiotic treatment, the recipient mice were orally administered prepared fecal suspensions every day for 21 consecutive days, with a gavage volume of 10 mL/kg. Specifically, mice in the ASD-FMT group received fecal suspensions derived from children with ASD, while those in the Control-FMT group were treated with fecal suspensions from healthy, typically developing children.

### 2.4. Behavioral Tests

Following 3 weeks of FMT treatment, behavioral tests were performed on 7-week-old mice from 9:00 a.m. to 5:00 p.m., with a minimum 24 h interval between each test. The following tests were conducted sequentially: open field test, three-chamber social interaction test, and marble-burying test. All testing apparatus were wiped with 75% ethanol between trials to eliminate olfactory cues. Behavioral testing and image analysis were completed by investigators blinded to group allocation.

#### 2.4.1. Open Field Test

The open-field test was used to evaluate spontaneous locomotor activity and anxiety-related exploratory behaviors in a novel environment. The testing arena was 40 cm × 40 cm, with a central zone measuring 20 cm × 20 cm. All mice were acclimated to the testing room for 30 min before formal testing. Each mouse was individually placed in the central region of the open field, and their spontaneous movements were continuously videotaped over a 10 min trial. Behavioral parameters, including total travel distance (mm), average moving speed, and the distance and time in the center zone, as well as the distance ratio (distance traveled in central area/total distance traveled in the open field), were subsequently quantified and analyzed.

#### 2.4.2. Three-Chamber Social Test

The three-chamber social interaction assay was performed using a 60 cm × 40 cm × 30 cm compartmentalized apparatus consisting of left, right, and central chambers. Test mice were initially placed into the central compartment to eliminate positional bias related to the order of entry into the lateral chambers. Prior to formal behavioral testing, each subject mouse underwent a 10 min free habituation period to explore the entire apparatus.

Following habituation, an age- and sex-matched unfamiliar mouse (Stranger 1) was confined within the left chamber, and the test mouse’s exploratory behaviors across all three compartments were video-recorded over a subsequent 10 min testing session. After completion of this phase, the entire arena was thoroughly cleaned with 75% ethanol to eliminate residual fecal debris and olfactory cues. For the social novelty phase, a second age- and sex-matched unfamiliar mouse (Stranger 2) was placed in the right chamber, whereas Stranger 1 remained housed in the left chamber; the test mouse was then monitored for a third 10 min period with continuous behavioral recording.

We quantified the cumulative time test mice spent interacting within the left (L) and right (R) chambers. To prevent scoring artifacts, direct social contact time toward the empty cup, for Stranger 1 or Stranger 2, was defined as the duration during which the subject’s head remained within 3 cm of the cup perimeter. The preference indices of each individual mouse were calculated: the social preference index = (Time_Stranger 1_ − Time_empty cup_)/(Time_Stranger 1_ + Time_empty cup_), and the social novelty index = (Time_Stranger 2_ − Time_Stranger 1_)/(Time_Stranger 2_ + Time_Stranger 1_).

Both Stranger 1 and Stranger 2 were age-matched wild-type (WT) male mice group-housed in independent home cages. These animals received daily 20 min habituation training inside the wire restraining cups for a minimum of three consecutive days before behavioral assessment.

#### 2.4.3. Marble-Burying Test

The marble-burying assay was conducted in rectangular test cages filled with sawdust bedding to a uniform depth of 5 cm. Twenty identical glass marbles were arranged atop the bedding in a 4-row × 5-column grid layout. Individual mice were gently placed at the cage center and permitted unrestricted exploratory activity for a 10 min testing period. Upon assay termination, the number of buried marbles was enumerated manually. A marble was scored as buried when over half of its total volume was covered by bedding material.

At the experimental endpoint, mice were euthanized using an overdose of sodium pentobarbital under deep anesthesia. Death was verified by permanent cessation of cardiac pulsation and spontaneous respiration, alongside fixed and dilated pupils. All tissue specimens were promptly harvested while animals remained under anesthesia to minimize postmortem ischemia and processing delay.

### 2.5. 16S rRNA Gene Sequencing and Sequence Analysis

Fecal samples were collected from Control-FMT and ASD-FMT mice and immediately stored at −80 °C. Total genomic DNA was extracted using the TIANamp Stool DNA Kit (DP712, TIANGEN, Beijing, China) following the manufacturer’s standard protocols. Purified DNA was quantified with a Qubit fluorometer (Invitrogen, Carlsbad, CA, USA) and stored at −20 °C for downstream analysis. The V3–V4 hypervariable region of the bacterial 16S rRNA gene was amplified using the primer pair 341F (5′-CCTACGGGNGGCWGCAG-3′) and 805R (5′-GACTACHVGGGTATCTAATCC-3′). Sample-specific Illumina index sequences were appended to both forward and reverse primers to enable multiplexed deep sequencing. PCR amplicons were purified using AMPure XP Beads (Beckman Coulter Genomics, Danvers, MA, USA) and quantified on a Qubit fluorometer (Invitrogen, USA). The quality of purified PCR products was further assessed with the Illumina library quantification kit (Kapa Biosciences, Woburn, MA, USA). The constructed amplicon library was subjected to paired-end sequencing (2 × 300 bp) on a DNBSEQ-G99 platform (LC-Bio Technology Co., Ltd., Hangzhou, China). Negative controls were strictly set up during both DNA extraction and PCR amplification to rule out potential environmental and reagent contamination throughout the experiment.

Raw paired-end sequencing data were processed with QIIME2 (v2025.7) [32]. Adapters and primer sequences were trimmed using cutadapt implemented in the QIIME2 cutadapt plugin. Forward and reverse reads were merged via the vsearch merge-pairs command within QIIME2 (v2025.7). Low-quality sequences were filtered according to quality-score thresholds using the q2-quality-filter q-score workflow. Chimeric sequences were detected and removed with vsearch. This workflow yielded high-quality, non-chimeric reads for subsequent analyses. Clean reads were then denoised to generate amplicon sequence variants (ASVs) using DADA2. ASVs with total counts ≤2 across all samples were discarded. Taxonomic classification of ASVs was performed in QIIME2 (v2025.7) using the Naive Bayes classifier against the SILVA database (release 138.2) [33], with a confidence cutoff of 0.7. Beta-diversity patterns of microbial community composition were explored via principal component analysis (PCA), where sample ordination was based on Euclidean distances derived from ASV feature tables. Group-level differences in microbiota structure were statistically evaluated using PERMANOVA (permutational multivariate analysis of variance) and ANOSIM (analysis of similarities) implemented in QIIME2 (v2025.7). Per-sample sequencing and quality control summary are shown in Supplementary Table S1.

### 2.6. Immunofluorescence (IF) Staining

The brain sections underwent 1 h room-temperature blocking in 0.5% bovine serum albumin (BSA) solution after three sequential phosphate-buffered saline (PBS) washes. Subsequently, tissue sections were incubated overnight at 4 °C with primary antibodies: mouse anti-GFAP monoclonal antibody (1:800, CST, Danvers, MA, USA) for astrocyte labeling, rabbit anti-Iba-1 monoclonal antibody (1:800, CST, Danvers, MA, USA) for microglia staining, mouse anti-c-Fos monoclonal antibody (1:500, Abcam, Cambridge, UK) for neuronal activation staining, and rabbit anti-DCX monoclonal antibody for hippocampal neurogenesis (1:500, Abcam, Cambridge, UK). After repeated PBS washes, sections were further incubated with corresponding secondary antibodies for 1 h at ambient temperature, including goat anti-rabbit IgG (1:500, CST, Danvers, MA, USA) for Iba-1 and DCX and goat anti-mouse IgG (1:500, CST, Danvers, MA, USA) for c-Fos detection.

Subsequently, slides were counterstained with 4′,6-diamidino-2-phenylindole (DAPI) for 5 min at room temperature prior to three PBS washes. Following coverslip mounting, stained specimens were promptly visualized under an ultra-high-resolution laser scanning confocal microscope (Zeiss, Oberkochen, Baden-Württemberg, Germany). Quantitative analysis of immunofluorescence signals was performed using ImageJ 1.52i software. The expression levels were quantified based on integrated optical density (IOD) and the corresponding stained pixel area (Area), with IOD values calculated from threshold-defined positive pixels within each captured image.

For quantitative statistics of whole-brain sections, three representative microscopic fields covering the CA1, CA3, and DG/CC regions were selected from the ipsilateral cerebral hemisphere (left and right). The mean density value was defined as the ratio of total IOD to stained pixel area (Mean density = IOD/Area).

### 2.7. Mass Spectrometry Assay for Data-Independent Acquisition (DIA) and Proteomics Data Handling

In brief, disulfide bonds in samples were reduced through incubation with dithiothreitol (DTT) at 37 °C for 1.5 h. Subsequently, iodoacetamide (IAA) was supplemented to block reduced cysteine residues, followed by a 30 min incubation at room temperature in the dark. Trypsin was then added at a trypsin-to-protein mass ratio of 1:50, and digestion proceeded overnight (15–18 h) at 37 °C. After enzymatic digestion, the resultant peptides were desalted using an MCX desalting column (omicsolution, OS-MCX-1ML), vacuum-dried, and reconstituted in 20 μL aqueous 0.1% (*v*/*v*) formic acid. Peptide concentration was quantified by measuring ultraviolet absorbance at 280 nm. For subsequent DIA analysis, indexed retention time (iRT) calibration peptides were spiked into the prepared peptide mixture prior to LC-MS measurement.

The peptides from each sample were analyzed by an OrbitrapTM AstralTM mass spectrometer (Thermo Scientific, Waltham, MA, USA) connected to a Vanquish Neo liquid chromatography system (Thermo Scientific, Waltham, MA, USA) in data-independent acquisition (DIA) mode. For MS1 acquisition, precursor ions were monitored across an *m*/*z* range of 380–980 with a resolution set to 240,000 at *m*/*z* 200, a normalized AGC target of 500%, and a maximum injection time (IT) of 5 ms. The MS2 DIA was configured with 299 sequential isolation windows of 2 *m*/*z* each. Fragmentation was performed via higher-energy collisional dissociation (HCD) at a normalized collision energy of 25 eV, with the normalized AGC target set to 500% and maximum IT limited to 3 ms.

IA raw data were analyzed using DIA-NN software (version 1.8.1) with the following optimized parameters: trypsin was specified as the digestion enzyme, with a maximum of one missed cleavage allowed. Carbamidomethylation on cysteine (C) was set as a fixed modification, while methionine oxidation (M) and protein *N*-terminal acetylation were defined as dynamic modifications. Protein identification was validated at a 99% confidence level with a false discovery rate (FDR) threshold of ≤1% for all results.

### 2.8. Mass Spectrometry Assay for Data-Independent Acquisition (DIA) and Phosphoproteomic Proteomic Data Handling

Hippocampal tissues were homogenized using an MP FastPrep-24 homogenizer (24 × 2 beads, 6.0 m/s, 60 s per cycle, and two cycles in total), followed by the addition of UA lysis buffer containing 8 M urea and 100 mM Tris-HCl (pH 8.5). Tissue lysates were optionally subjected to sonication. Following centrifugation at 14,000× *g* for 40 min, the resulting supernatants were quantified for total protein concentration using a BCA Protein Assay Kit. An equal amount of 15 μg protein from each sample was blended with 5× protein loading buffer and denatured by boiling for 5 min. Subsequently, proteins were separated via 4–20% gradient SDS-PAGE under a constant voltage of 180 V for 45 min, and resolved protein bands were stained with Coomassie blue R-250 for visualization.

DTT was supplemented to a final concentration of 20 mM, followed by shaking at 600 rpm for 1 min and incubation at 37 °C for 1.5 h. After cooling to ambient temperature, 20 mM IAA was added to alkylate free cysteines, and samples were incubated in the dark for 30 min. Urea was diluted below 1.5 M using 450 μL of 100 mM NH_4_HCO_3_, and trypsin was introduced at a trypsin/protein mass ratio of 3:200 for overnight digestion (15–18 h, 37 °C). Digestion was terminated by acidification to pH 3 with sequential addition of 0.01% TFA and 10% TFA. The resulting peptides were desalted with Empore™ C18 SPE cartridges (30 μm, Waters, Milford, MA, USA) and vacuum-lyophilized.

Phosphopeptide enrichment was performed with the High-Select™ Fe-NTA Phosphopeptide Enrichment Kit (Thermo Scientific, Waltham, MA, USA) following the manufacturer’s protocol. After lyophilization, enriched phosphopeptides were reconstituted in 20 μL of loading buffer containing 0.1% formic acid.

Indexed retention time (iRT) calibrant peptides were spiked into 2 μg phosphopeptides per sample prior to LC-DIA measurement. Separation was achieved on a Vanquish Neo UHPLC system coupled to an Orbitrap™ Astral™ mass spectrometer (Thermo Scientific, Waltham, MA, USA) in the data-independent acquisition (DIA) mode. MS1 scans covered *m*/*z* 380–980 at a resolution of 240,000 (at *m*/*z* 200), with a normalized AGC target of 500% and maximum injection time (IT) of 5 ms. DIA-MS2 acquisition employed 300 isolation windows of 2 *m*/*z*; peptides were fragmented by HCD at 25 eV under 300% normalized AGC and 3 ms maximum IT.

DIA raw data were processed using Spectronaut with the following parameters: trypsin as the digestive enzyme and up to two missed cleavages permitted. Carbamidomethylation at cysteine (C) was defined as a fixed modification, whereas methionine oxidation (M) and protein *N*-terminal acetylation were set as variable modifications. Peptide and protein identifications were filtered at a false discovery rate (FDR) ≤ 1%.

### 2.9. Bioinformatic Analysis

Hierarchical clustering was conducted via Cluster 3.0 and Java TreeView (http:/jtreeview.sourceforge.net (accessed on 30 September 2025)). Differentially expressed protein sequences were annotated for GO using NCBI BLAST+client software (ncbi-blast-2.2.28+-win32.exe), InterProScan 5.64-96.0, and Blast2GO Pipeline 2.5.0, with GO visualization accomplished by R scripts. KEGG pathway enrichment was performed by sequence alignment against the online KEGG database. Gene set enrichment analysis (GSEA) was implemented on ranked proteins sorted by fold change (FC); R clusterProfiler (v4.4.4) was used with a *p*-value cutoff of 0.05. Phosphorylation-flanking sequences (13 residues centered on modification sites) were subjected to motif prediction using MEME 5.0.2 (width = 13, maximum occurrences = 20, and species-specific background). Following normalization to total peak intensity, datasets were Pareto-scaled and analyzed by principal component analysis (PCA) in SIMCA-P 14.1.

### 2.10. Golgi Staining

Whole brain tissues from each group were collected and subjected to Golgi–Cox staining using a Golgi Stain Kit (Servicebio, Wuhan, China). Briefly, the brain samples were fixed in Golgi solution and stained with Golgi–Cox working solution for 14 days at room temperature in a ventilated, dark, and cool environment. Post-staining, tissues were treated with kit-provided processing solution for 1 h, followed by medium replacement and a 3-day dark incubation at 4 °C. The coronal brain slices (thickness: 60 μm) were cut on a Leica cryostat microtome. The sections were washed with ultrapure water and immersed in Golgi developer for 30 min. Specimens were imaged with a digital slide scanner, digitally scanned, and analyzed with ImageJ 1.52i software.

### 2.11. Western Blot Analysis

Total protein was extracted in RIPA buffer containing a protease inhibitor cocktail, and sample protein concentration was determined using a BCA assay kit. Equal protein loads were fractionated by SDS-PAGE before being transferred to PVDF membranes. Membranes were blocked in 5% BSA for 1 h at room temperature and then incubated overnight at 4 °C with primary antibodies against ZO-1 (1:3000, Abcam, Cambridge, UK), Occludin-1 (1:1000, Servicebio, Wuhan, China), Claudin-5 (1:1000, Servicebio, Wuhan, China), FNDC3A (1:1000, Proteintech, Wuhan, China), PSD-95 (1:2000, Signalway Antibody, College Park, MD, USA), SYP (1:1000, Servicebio, Wuhan, China), and β-actin (1:4000, Proteintech, Wuhan, China). Post-wash steps were followed by 1.5 h incubation with HRP-linked secondary antibody (1:5000, MCE, Monmouth Junction, NJ, USA) at room temperature. Immunoreactive bands were detected with Super ECL Plus and documented by gel imaging equipment. To ensure technical rigor, Western blot validation was performed using *n* = 3 distinct biological mouse samples per group, with each biological sample being run in technical triplicates across independent gel runs.

### 2.12. Unphosphorylated and Phosphorylated Structure Predictions

Structures of both unphosphorylated and phosphorylated protein forms were predicted using the AlphaFold3 server v0.1.30 (https://alphafoldserver.com/) and an input JSON file using standard modification tags (e.g., defining S482 as SEP [phosphoserine]).

Structural visualization was carried out with PyMOL 3.1.0 software. The AlphaFold3 predictions were based on unmodified canonical protein sequences obtained from UniProtKB. To assess the conformational heterogeneity of SHANK3 and SRRM2 phosphoproteins, individual protein structural conformers were clustered based on root mean square deviation (RMSD) metric values.

### 2.13. Molecular Docking

HADDOCK 2.4 webserver (build 2024.02) (https://rascar.science.uu.nl/HADDOCK2.4/ (accessed on 20 May 2026)) was used to perform in silico docking of SHANK3 to the PSD-95–Homer1 complex [34,35]. Rigid-body docking, semi-flexible refinement, and water refinement are the steps in this data-driven docking approach. The weighted sum (HADDOCK scores) was calculated from weighted van der Waals, electrostatic, desolvation, restraint energies, and buried surface area and was used to rank the resulting clusters. All computational parameters on the HADDOCK 2.4 web server were retained at default values and are shown in Supplementary Table S4. The fraction of common contacts (FCC), a measure of the similarity of intermolecular contact patterns, was used to cluster produced protein–protein binding poses after docking calculations. Final docked structures were clustered by the FCC with a threshold of 0.6 and a minimum cluster size of 4 [36].

### 2.14. ELISA

The human FNDC3A ELISA Kit (Kanglang Biological, Shanghai, China) was based on a sandwich enzyme-linked immunosorbent assay (sandwich ELISA). Capture antibodies were pre-immobilized onto 96-well microplates, while biotin-labeled antibodies served as detection antibodies. After sequential addition of calibrators, test plasma samples (children with ASD, *n* = 20; control children, *n* = 20) and biotinylated detection antibodies into plate wells, excess unbound reagents were removed via washing with washing buffer. Horseradish peroxidase-conjugated streptavidin (HRP-Streptavidin) was then supplemented, followed by another wash step to eliminate unbound conjugates. The chromogenic substrate TMB was applied to initiate the HRP-dependent colorimetric reaction: HRP catalyzed TMB to form a blue intermediate, which turned yellow upon addition of acidic stop solution. The absorbance intensity of the yellow product at 450 nm directly correlates with the quantity of captured FNDC3A antigen in each well. Optical density (OD) values were measured using a microplate spectrophotometer at 450 nm, and target protein concentrations were calculated from the standard curve.

### 2.15. Statistical Analysis

Data were expressed as the mean ± S.E.M. Statistical analyses were performed using GraphPad Prism 10.0.1 software (GraphPad Software, Inc.); pairwise group comparisons were achieved using Student’s *t*-test, whereas the nonparametric Kruskal–Wallis test was adopted for non-normally distributed data. Spearman’s rank correlation analysis was utilized to assess inter-association among measured parameters using the OmicStudio tools at https://www.omicstudio.cn/too (accessed on 15 April 2026). Statistical significance was set at *p* < 0.05.

## 3. Results

### 3.1. FMT-Induced Gut Microbial Alterations and Autism-like Behaviors in Recipient Mice

**Figure 1:**
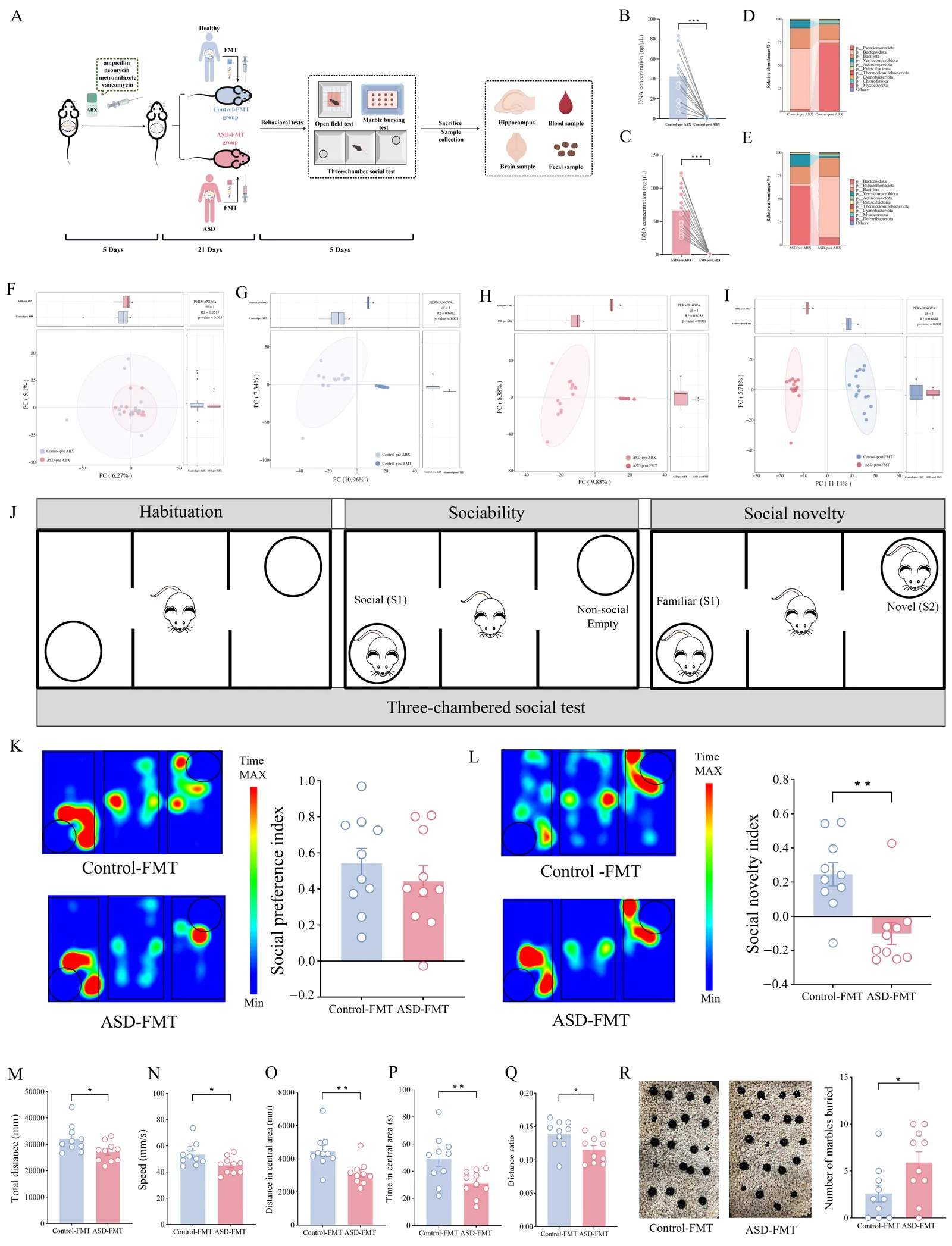
The microbiota profiles from 16S rRNA gene sequencing and behavioral alterations. (**A**) Schematic diagram of the FMT procedure. (**B**–**I**) 16S rRNA sequencing analyses across experimental groups. ASD-pre ABX group: ASD group prior to ABX administration; ASD-post ABX: ASD group after ABX administration; ASD-post FMT: ASD group after FMT; Control-pre ABX group: Control group prior to ABX administration; Control-post ABX: Control group after ABX administration; Control-post FMT: Control group after FMT. (**B**,**C**) Fecal bacterial DNA concentrations. (**D**,**E**) Stacked bar chart plots illustrating gut microbiota composition at the phylum level in mice before and after ABX administration. (**F**) Principal component analysis (PCA) of gut microbiota composition for Control-pre ABX and ASD-pre ABX group (*n* = 15 per group). Different lowercase letters denote statistically significant differences between groups (*p* < 0.05); groups sharing identical letters are not significantly different. (**G**) PAC of gut microbiota profiles for Control-pre ABX and Control-post FMT group (*n* = 15 per group). (**H**) PAC of gut microbiota profiles for ASD-pre ABX and ASD-post FMT group (*n* = 15 per group). (**I**) PAC of gut microbiota profiles for Control-post FMT group and ASD-post FMT group (*n* = 15 per group). (**J**–**R**) Behavioral alterations in mice following fecal microbiota transplantation (FMT) from children with ASD (the ASD-post FMT group, hereinafter referred to as the ASD-FMT group) and typically developing (TD) controls (Control-post FMT group, hereinafter referred to as the Control-FMT group). Animals were assigned to behavioral (*n* = 10), omics (*n* = 5), and molecular/histological validation cohorts (10 animals in total). No animals were arbitrarily excluded. (**J**) Experimental workflow of the three-chamber social interaction test. (**K**) Social preference index, reflecting the preference of mice for social interaction with a conspecific stranger mouse (S1) relative to an empty cup. (**L**) Social novelty index, representing the preference of mice for a novel unfamiliar conspecific (S2) versus a familiar conspecific (S1). (**M**) Total movement distance of mice in the open field test. (**N**) Average movement speed of mice in the open field. (**O**) Travel distance in the central area of the open field. (**P**) Time spent in the central area of the open field. (**Q**) The distance ratio = distance traveled in the central area/total distance traveled in the open field. (**R**) Representative images and quantitative analysis of buried marbles in the marble-burying test after FMT (*n* = 10 mice per group). Statistical significance was determined using the unpaired Student’s *t*-test. * *p* < 0.05, ** *p* < 0.01, *** *p* < 0.001.

The schematic workflow of the FMT procedure was shown in Figure 1A. To assess the change in gut microbiota, we quantified the total fecal bacterial DNA concentration and performed 16S rRNA sequencing on fecal samples collected from mice at three time points: prior to ABX administration, post-ABX administration, and after FMT. We observed a marked reduction in fecal bacterial DNA concentration following ABX administration (Figure 1B,C). The sequencing results revealed substantial simplification of the gut microbiota following ABX exposure. The microbial community transitioned from a highly diverse assemblage dominated by Bacteroidota and Bacillota toward a Pseudomonadota-enriched community (Figure 1D,E). These results indicate that ABX administration induces major depletion of the gut microbiota. In addition, the principal component analysis (PCA) ordination based on Euclidean distance revealed substantial overlap between samples from the Control and ASD group prior to ABX exposure (Figure 1F). For the control group, PCA showed a complete division of gut microbiota profiles collected before ABX administration and after FMT administration (Figure 1G). Comparable results were observed in the ASD group (Figure 1H). PCA-based dissimilarity analysis uncovered clear separation between the Control and ASD groups after FMT, indicative of distinct gut microbiota compositions (Figure 1I). PERMANOVA analyses further confirmed significant differences in overall microbial community structure between the two groups (*p* = 0.001).

To investigate the contribution of the gut microbiota derived from children with ASD to the mouse behavior, a battery of behavioral assays was conducted, including the open field test, three-chamber social test and marble-burying test (Figure 1A). The impacts of ASD-FMT on social behavior were assessed using a well-established three-chamber social test (Figure 1J). In the sociability test (Figure 1K), ASD-FMT mice showed reduced social contact duration with Stranger 1 relative to the empty cup, yet the difference failed to reach statistical significance. The result indicated that there is an unobvious change in sociability. Remarkably, during the social novelty phase, Control-FMT mice exhibited longer interaction time with the novel unfamiliar mouse (Stranger 2), which yielded a positive social novelty index (Figure 1L). In contrast, ASD-FMT mice exhibited a significantly reduced time interacting with the novel unfamiliar mouse (Stranger 2), which led to a negative value for the social novelty index, indicating impaired social novelty preference (Figure 1L). The significant difference in social novelty behaviors was observed between the ASD-FMT and Control-FMT groups in the three-chamber social assay. It follows that the primary social deficit was specifically restricted to social novelty preference (Stranger 2 vs. Stranger 1), while sociability (Stranger 1 vs. empty cup) did not reach statistical significance.

In the open field test, significant disparities in locomotor activity and exploratory behaviors were observed between ASD-FMT and Control-FMT groups (Figure 1M–Q). ASD-FMT mice displayed a significant decline in total moving distance and average speed in the entire field over 10 min, compared with the Control-FMT group (*p* < 0.05) (Figure 1M,N). The time, distance, and distance ratios (distance traveled in central area/total distance traveled in the open field) of the ASD-FMT group were significantly reduced in the center zone compared with those of the Control-FMT group (*p* < 0.05) (Figure 1O–Q). The reduced total locomotor activity and average speed in the open field test could influence exploration time in both the center zone and social chambers. To minimize the confounding impact of reduced total locomotor activity and movement speed in ASD-FMT mice on social behavioral outputs and center-zone exploration, we calculated normalized preference indices and distance ratios for statistical testing, rather than relying on raw residence time and raw travel distance values.

Additionally, in the marble-burying test, the number of buried marbles was notably higher in the ASD-FMT group (Figure 1R), which implies that gut microbiota from children with ASD contributes to repetitive stereotyped actions. To summarize, transplantation of gut microbiota derived from children with ASD into wild-type mice recapitulated several ASD-like behavioral phenotypes, including social novelty deficits, hypoactivity, anxiety-related behavior, and repetitive stereotypies.

### 3.2. ASD-FMT Elicited Glial and Neuronal Hyperactivation While Suppressing Neurogenesis in Recipient Mice

**Figure 2:**
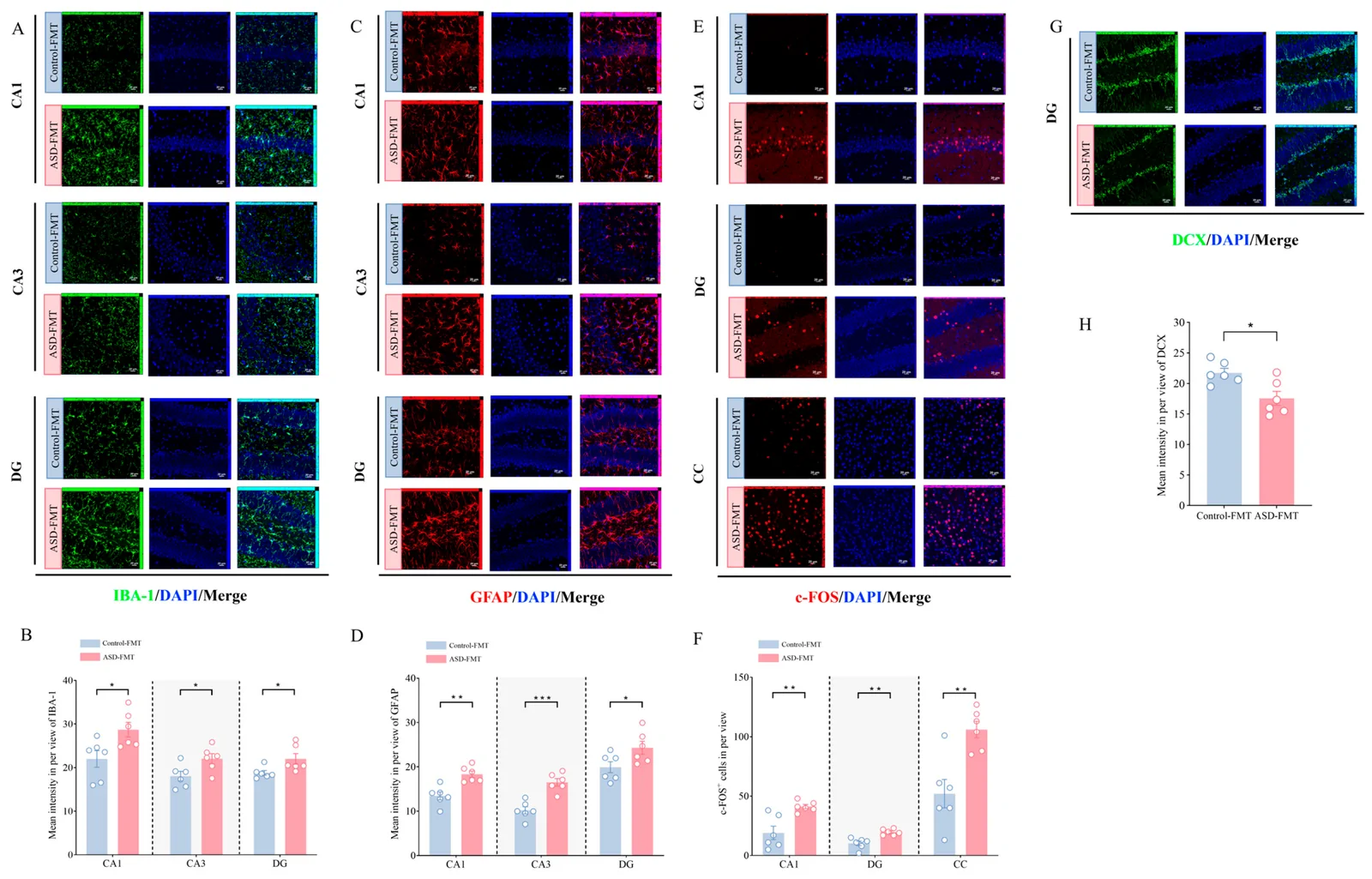
Immunofluorescence staining analyses of biomarker expression in the hippocampus of mice in the ASD-FMT and Control-FMT groups. (**A**) Representative immunofluorescence images of IBA-1 staining in the mouse hippocampus from the ASD-FMT and Control-FMT groups. Scale bar: 20 µm. (**B**) Quantitative analysis of IBA-1 fluorescence intensity based on IF staining. (**C**) Representative immunofluorescence images of GFAP staining in the mouse hippocampus from the ASD-FMT and Control-FMT groups. Scale bar: 20 µm. (**D**) Quantitative analysis of GFAP fluorescence intensity based on immunofluorescence staining. (**E**) Representative immunofluorescence images of C-FOS staining in the mouse hippocampus from the ASD-FMT and Control-FMT groups. Scale bar: 20 µm. (**F**) Quantitative analysis of the number of c-FOS-positive cells based on immunofluorescence staining. (**G**) Representative immunofluorescence images of DCX staining in the mouse hippocampus from the ASD-FMT and Control-FMT groups. Scale bar: 20 µm. (**H**) Quantitative analysis of DCX fluorescence intensity based on immunofluorescence staining. Statistical significance was determined using the unpaired Student’s *t*-test. * *p* < 0.05, ** *p* < 0.01, *** *p* < 0.001.

We further conducted IF staining to explore the potential effects of gut microbiota from children with ASD on hippocampal glial cells and neurons. The IF staining results revealed that ASD-FMT markedly activated microglia and astrocytes in the hippocampal CA1, CA2, and DG regions of the recipient mice (Figure 2A,C), as evidenced by elevated fluorescence intensity of IBA1- and GFAP-positive cells (Figure 2B,D). We also performed IF staining to detect c-Fos, which serves as a marker of neuronal activation (Figure 2E). Compared with the Control-FMT group, the ASD-FMT group displays excessive neuronal activation, as evidenced by a marked increase in the number of c-Fos-positive neurons in the hippocampal CA1, DG, and cerebral cortex (CC) regions of the recipient mice (Figure 2F). Next, we examined neurogenesis in the DG regions and detected a decrease in the fluorescence intensity of positive cells labeled by the immature neuron marker doublecortin (DCX) (Figure 2G,H). These results revealed aberrant activation of microglia, astrocytes, and neurons, as well as disrupted neurogenesis.

### 3.3. Limited Differential Protein Expression Following ASD-FMT

**Figure 3:**
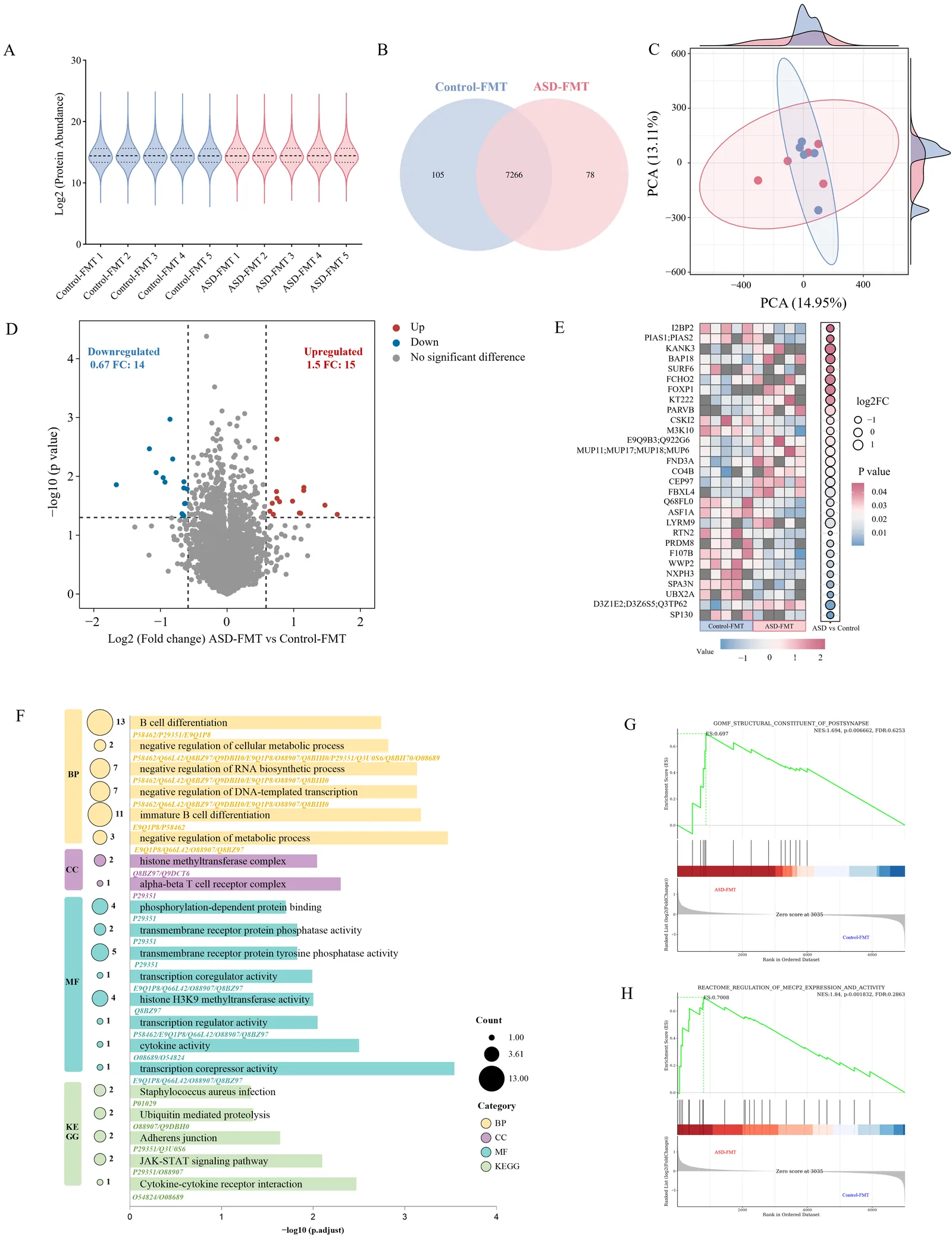
Global proteomic profiling of hippocampal tissues from mice in the ASD-FMT and Control-FMT groups. (**A**) Violin plots showing the overall distribution of Log2-transformed label-free quantification (LFQ) values across the global proteomic dataset. (**B**) Venn diagram illustrating the overlap of identified proteins between the ASD-FMT and Control-FMT groups. (**C**) Two-dimensional principal component analysis (PCA) revealing the overall proteomic differences between the ASD-FMT and Control-FMT groups. (**D**) Volcano plot displaying the fold changes and statistical significance of differentially expressed proteins (DEPs) in the ASD-FMT group relative to the Control-FMT group. The x-axis represents Log2 (ASD-FMT group/Control-FMT group) fold change, and the y-axis indicates −Log10 (*p*-value). Proteins with FC > 1.5 and *p* < 0.05 were defined as upregulated, while those with FC < 0.67 and *p* < 0.05 were defined as downregulated. The vertical dashed lines correspond to fold change = −0.67 and 1.5, and the horizontal dashed line indicates −log_10_(*p*-value) = 1 (*p* = 0.05). (**E**) Hierarchical clustering heatmap of DEPs identified between the ASD-FMT and Control-FMT groups. (**F**) Gene Ontology (GO) and Kyoto Encyclopedia of Genes and Genomes (KEGG) pathway enrichment analyses of the DEPs. (**G**,**H**) Gene Set Enrichment Analysis (GSEA) of proteomic data identifying representative ASD-related gene sets. (**G**) Structural constituent of postsynapse; (**H**) Regulation of MECP2 expression and activity.

Based on the obtained behavioral phenotypes and IF staining results, we proceeded to investigate global proteome expression variations in the hippocampus after ASD-FMT using proteomics. Our global proteome analysis showed consistent protein abundance distributions across all samples, as reflected by the log_2_-transformed values for all quantified proteins (*n* = 5 per group) (Figure 3A), which indicated remarkable sample consistency and biological reproducibility. The Venn diagram shows the intersection of protein number between ASD-FMT and Control-FMT groups in this study. A total of 7371 and 7344 proteins were identified in the Control-FMT and ASD-FMT groups, respectively (Figure 3B). Among these identified proteins, 7266 were common to both groups (Figure 3B), while 105 and 78 proteins were uniquely present in the Control-FMT and ASD-FMT groups, respectively (Figure 3B). PCA showed that the ASD-FMT group exhibited no obvious separation from Control-FMT group (Figure 3C). The identified proteins were further screened for differences, as shown in the volcano map (Figure 3D) and hierarchically clustered heatmap (Figure 3E). Proteins with an FC > 1.5 were defined as significantly upregulated, and those with an FC < 0.67 were deemed significantly downregulated at *p* < 0.05. Compared with the control group, 14 proteins were significantly upregulated and 15 were downregulated in the ASD-FMT group (Figure 3D,E). We found that multiple proteins associated with ASD and neurodevelopment, including FOXP1 [37,38], FBXL4 [39], and FNDC3A [40,41], showed significant differences between the ASD-FMT and Control-FMT groups (Figure 3E). GO and KEGG pathway analyses revealed significant enrichment in B cell differentiation, phosphorylation-dependent protein-binding, *Staphylococcus aureus* infection, and other pathways (Figure 3F). Proteomic GSEA revealed reduced postsynaptic structural constituents and dysregulated MECP2 expression and activity in mice administered ASD-derived FMT (Figure 3G,H). Accordingly, only 29 proteins had significant expression changes, and only a few correlated strongly with autism-associated proteins and pathways. Additionally, given the disrupted phosphorylation-dependent protein-binding pathway induced by ASD-FMT, we further explore changes in protein phosphorylation in the hippocampus of recipient mice.

### 3.4. Substantial Differential Phosphoprotein and Phosphopeptide Expression Following ASD-FMT

**Figure 4:**
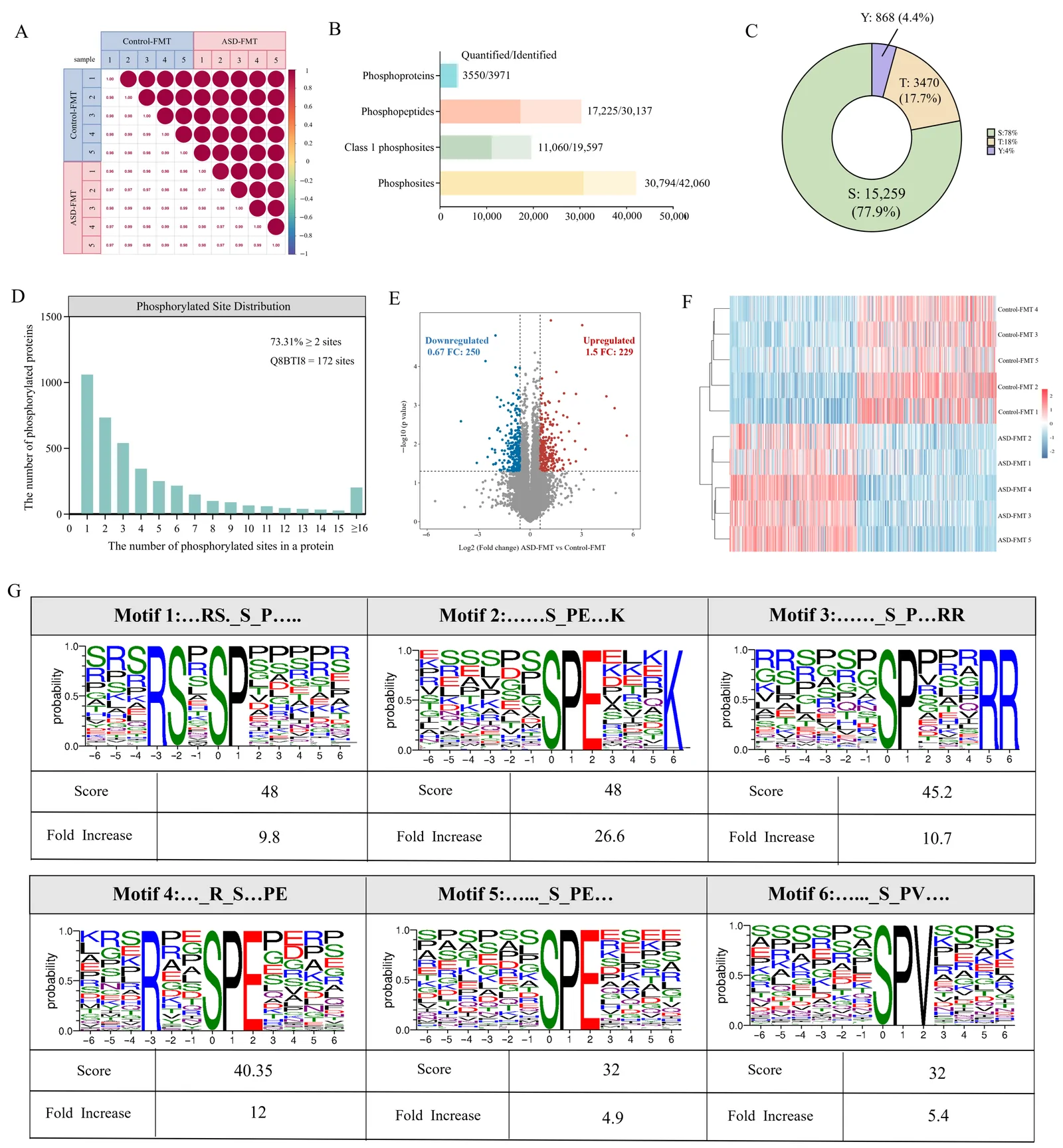
Global phosphoproteomic profiling of hippocampal tissues from mice in the ASD-FMT and Control-FMT groups. (**A**) Pearson correlation analysis of quantified phosphorylation sites across samples (*n* = 5 per group). (**B**) Summary statistics for the overall quantified and identified phosphoproteomic data. (**C**) Relative proportions of phosphoserine, phosphothreonine, and phosphotyrosine modification sites. (**D**) The number of phosphorylation modification sites in a protein. (**E**) Volcano plot illustrating the fold changes and statistical significance of differentially expressed phosphopeptides in the ASD-FMT group versus the Control-FMT group. The x-axis shows the Log2 (ASD-FMT group/Control-FMT group) fold change, and the y-axis represents −Log10 (*p*-value). Phosphopeptides with FC > 1.5 and *p* < 0.05 were considered significantly upregulated, whereas those with FC < 0.67 and *p* < 0.05 were considered significantly downregulated. The vertical dashed lines correspond to fold change = −0.67 and 1.5, and the horizontal dashed line indicates −log_10_(*p*-value) = 1 (*p* = 0.05). (**F**) Hierarchical clustering heatmap of differentially expressed phosphopeptides between the two groups. (**G**) Representative consensus phosphorylation motifs identified via the Motif-X algorithm. Motifs with *p* < 1 × 10^−6^ were screened, with corresponding motif scores and fold changes listed in the attached Table.

An Orbitrap Astral mass spectrometer coupled with data-independent acquisition (DIA) was utilized to profile hippocampal phosphoproteomes of mice in ASD-FMT (*n* = 5) and Control-FMT groups (*n* = 5). In the Control-FMT group, the numbers of identified class I phosphosites (phosphosites with probability > 0.75) in the hippocampus were 10,413, 10,996, 11,002, 11,160, and 11,082, and the corresponding numbers in the ASD-FMT group were 10,895, 10,926, 10,870, 10,874, and 10,947. After phosphorylation, site data were normalized via z-score transformation, and Pearson correlation analysis was conducted. All correlation coefficients exceeded 0.95 (Figure 4A), confirming favorable experimental reproducibility and compliance with quantitative quality control criteria [42,43,44]. In this study, the phosphoproteomic dataset yielded a total of 30,137 phosphopeptides mapped to 3971 proteins, as well as 19,597 class I phosphosites (phosphosites with probability > 0.75) and 42,060 phosphosites in this study (Figure 4B). The 19,597 quantified phosphopeptides contained 15,259 phosphoserine sites (77.9%), 3470 phosphothreonine sites (17.7%), and 868 phosphotyrosine sites (4.4%) (Figure 4C). It is noteworthy that the phosphoprotein SRRM2 (Q8BTI8) contained up to 172 phosphosites (Figure 4D). As reported by Silvestre Cuinat et al., 22 patients with loss-of-function variants in SRRM2 manifested mild developmental delay, predominant speech delay, autistic or attention-deficit/hyperactivity disorder features, and so on [31]. Differentially expressed phosphopeptides were visualized in volcano plots and hierarchically clustered heatmaps (Figure 4E,F). Phosphorylated peptides exhibiting FC > 1.5 were considered significantly upregulated, whereas those with FC < 0.67 were defined as significantly downregulated, with a threshold of *p* < 0.05 for statistical significance. The results indicated that gut microbiota derived from children with ASD exerted a notable effect on hippocampal phosphorylated peptides (Figure 4E,F), with 479 peptides showing significant changes. Among these peptides, 229 displayed elevated expression levels while 250 presented reduced phosphorylation abundance (Figure 4E). This means that phosphoproteomic profiles are more substantially modulated by gut microbiota derived from children with ASD, compared with proteomic characteristics. The specificity of kinase substrates relies on characteristic sequence motifs adjacent to phosphorylation loci, and distinct phosphorylation motifs generally correspond to different kinases [45]. Protein phosphorylation analysis identifies adjacent sequence motifs by analyzing amino acid residues flanking phosphorylation sites. We applied MEME software to analyze sequences spanning six amino acids upstream and downstream of the identified phosphorylation sites of serine residues. The top six hub motifs were filtered out in light of motif scoring values (Figure 4G). Residues of arginine (R), proline (P), and glutamate (E) exhibited preferential distribution adjacent to serine phosphorylation loci. The results revealed that the arginine-directed serine motif ranked the most prevalent among enriched consensus sequences.

### 3.5. Differential Phosphoproteins Enriched in Synapse-Related Pathways via GO and KEGG Analysis and Autistic Gut Microbiota Reduced Synaptic Density and Protein Expression

**Figure 5:**
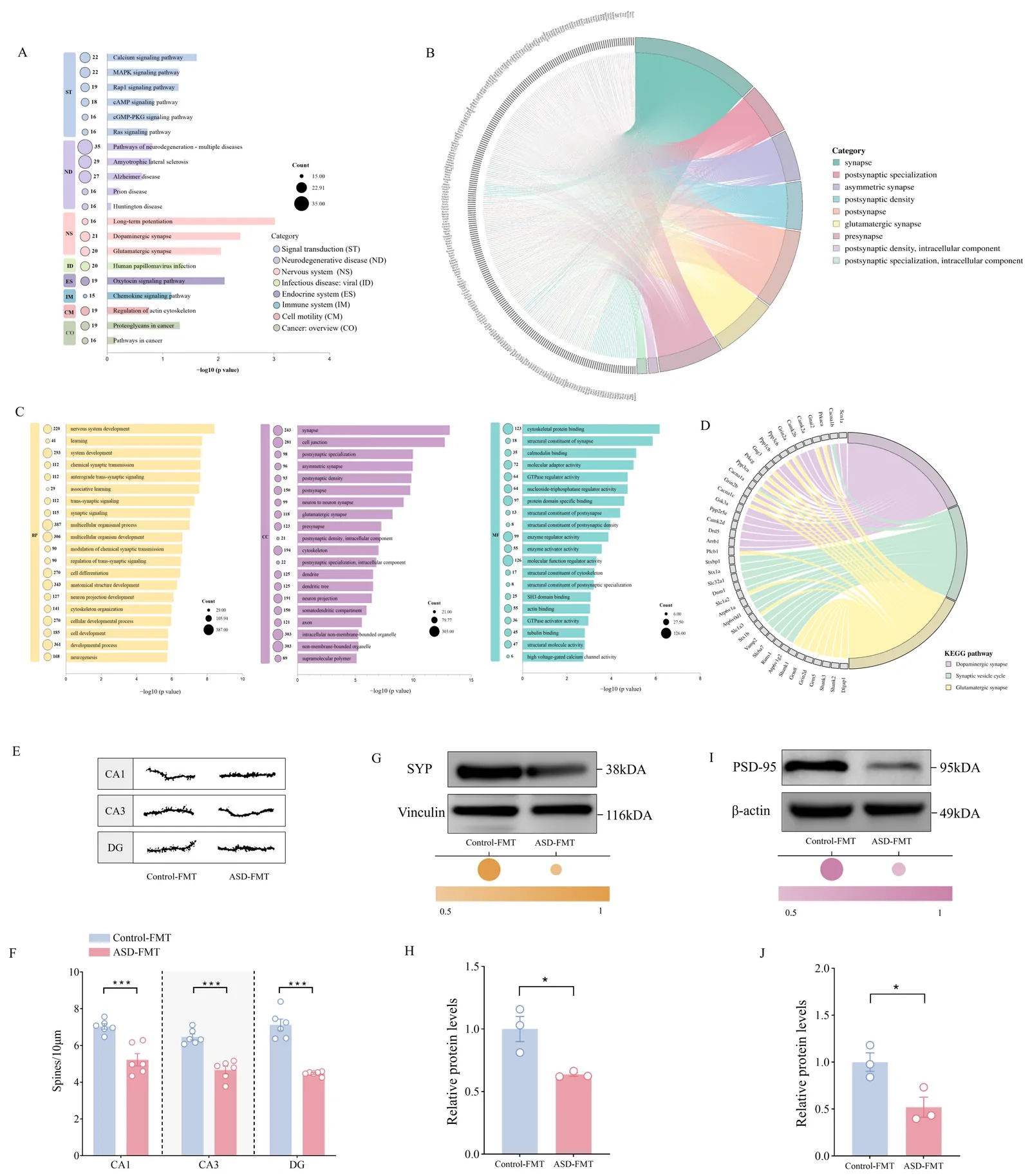
Aberrant synapse-associated pathways observed via phosphoproteomic KEGG/GO enrichment analyses, Golgi staining, and Western blot assays in ASD-FMT and Control-FMT mice. (**A**) Top 20 enriched level-2 KEGG pathways of differentially phosphorylated proteins. (**B**) Circle chord diagram visualizing the correlations between differentially phosphorylated proteins and representative synapse-related pathways. (**C**) Top 20 enriched pathways of differentially phosphorylated proteins in three GO categories, including biological process (BP), cellular component (CC), and molecular function (MF). (**D**) Circle chord diagram illustrating the associations between differentially phosphorylated proteins and screened synapse-related pathways. (**E**) Representative Golgi staining images of hippocampal neurons in the two groups. (**F**) Quantitative analysis of dendritic spine density in hippocampal neurons of the ASD-FMT and Control-FMT groups. Data are presented as means ± SEM (*n* = 3 per group). (**G**–**J**) Representative Western blot images and quantitative statistical analysis of the synaptic markers SYP and PSD-95 in hippocampal tissues from the two groups. Data represent n=3 biological replicates per group, with each sample analyzed in technical triplicate across independent experiments. Statistical significance: * *p* < 0.05, *** *p* < 0.001.

To further explore pathways related to differentially phosphorylated proteins and clarify their potential biological functions, we conducted GO and KEGG pathway analyses. The top 20 enrichment pathways identified using KEGG analysis (level 2) showed that the differentially phosphorylated proteins were predominantly enriched in neurodegeneration from multiple diseases and synapse-related pathways (Figure 5A,B). We then analyzed the top 20 enriched pathways of GO enrichment analysis, which were classified into biological process, cellular component, and molecular function (Figure 5C,D). Consistently, GO analysis revealed a high degree of enrichment in nervous system development in biological processes, synapse, postsynaptic density, and postsynapse and presynapse in cellular components, as well as structural constituent of postsynapse in molecular functions, and so on (Figure 5C,D). Collectively, these findings indicate that hippocampal differentially phosphorylated proteins are primarily linked to nervous system development and synapse-related pathways. Notably, these pathway changes emerged after ASD-FMT, suggesting that differentially phosphorylated proteins triggered by gut microbiota derived from children with ASD may exert prominent effects on hippocampal synapses.

For further verification, Golgi staining was implemented to assess hippocampal synaptic quantity and density in the recipient mice of ASD-FMT and Control-FMT groups (Figure 5E). A remarkable decline in dendritic spine density of hippocampal neurons was detected in the ASD-FMT group compared with the Control-FMT group (Figure 5F). Additionally, Western blot analysis was adopted to quantify the expression of SYP and the postsynaptic protein PSD-95, so as to corroborate the adverse impact of gut microbiota derived from children with ASD on hippocampal synaptic function. Western blot results showed that the expression levels of SYP and PSD-95 in the hippocampus were obviously decreased in the ASD-FMT group compared with the Control-FMT group (Figure 5G–J). Collectively, these findings demonstrate that gut microbiota from children with ASD significantly reduces synaptic density and related protein expression, thereby potentially contributing to hippocampal synaptic dysfunction via abnormal phosphorylation.

### 3.6. ASD-FMT Suppresses the Expression of BBB Tight Junction Proteins in Recipient Mice

**Figure 6:**
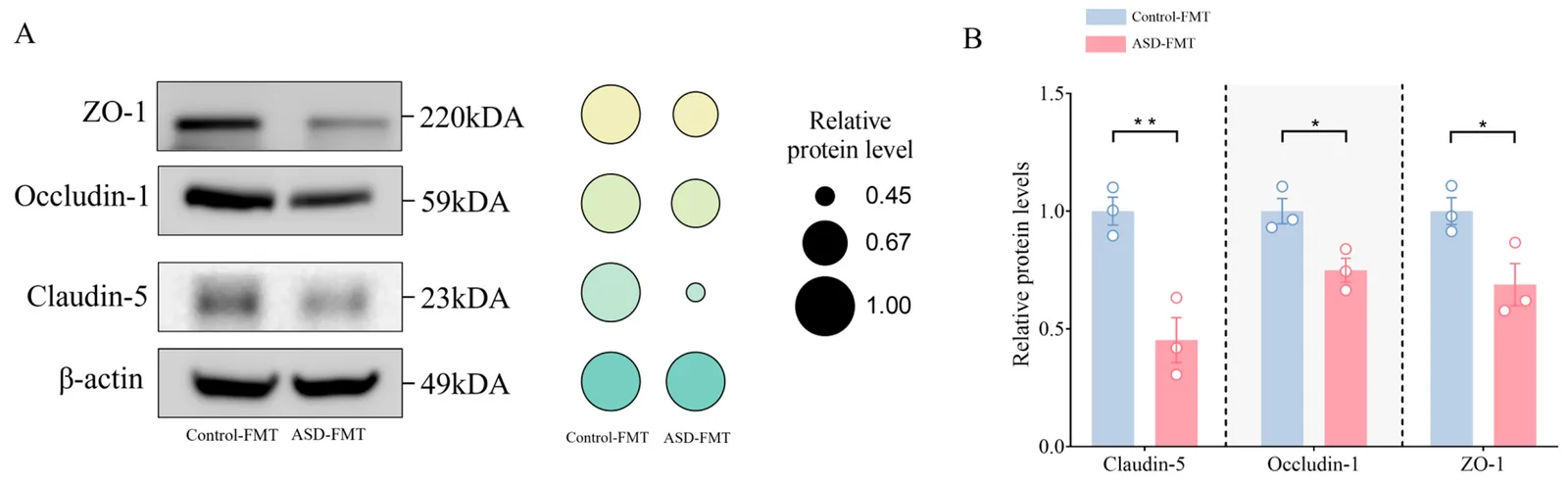
Expression levels and quantitative analysis of ZO-1, Occludin-1, and Claudin-5 in the hippocampus of ASD-FMT and Control-FMT mice. (**A**) Representative Western blot images of ZO-1, Occludin-1, and Claudin-5 protein expression. (**B**) Quantitative analysis of the relative protein levels of ZO-1, Occludin-1, and Claudin-5 in hippocampal tissues. Data represent n=3 biological replicates per group, with each sample analyzed in technical triplicate across independent experiments. Protein expression levels were normalized to the internal reference β-actin. Data are presented as means ± SEM. * *p* < 0.05, ** *p* < 0.01.

We also found MAPK signaling, Rap1 signaling, calcium signaling, and cAMP signaling pathways enriched in the top 20 KEGG (level 2) analysis (Figure 5A). Accumulated evidence indicates the regulatory effects of these pathways on the blood–brain barrier [46,47,48,49]. Accordingly, to explore whether gut microbiota in children with ASD influences the BBB, we detected key tight junction proteins in the hippocampus using Western blotting. The results of Western blot analysis indicated that the hippocampus of the ASD-FMT group exhibited notably reduced expression of ZO-1, Occluding-1, and Claudin-5 compared with the Control-FMT group (Figure 6A,B). Collectively, gut microbiota derived from children with ASD may induce the potential impairment of blood–brain barrier integrity.

### 3.7. Aberrant Phosphorylation Induced by ASD-FMT Can Change Protein Conformation and Impair Binding Affinity, as Exemplified by SHANK3 and SRRM2

Functional state transitions are commonly modulated by post-translational modifications such as phosphorylation, which dynamically alter protein structure, folding, function, and intermolecular interactions [17]. Recent breakthroughs in deep learning, represented by AlphaFold, have revolutionized protein structure prediction and achieved accuracy comparable to experimental measurements [17,50]. We adopted the circular chord diagram above to illustrate the relationships between selected synapse-related pathways and differentially phosphorylated proteins (Figure 5B,D). It is worth noting that differentially phosphorylated SHANK3 protein was detected in synapse-related pathways. The Shank3 gene encodes a synaptic scaffolding protein, and existing studies have confirmed that SHANK3 exerts pivotal effects on synaptic structure and function [51]. Disruptions in SHANK3 contribute to synaptic dysfunction, a prominent pathological hallmark of ASD [52]. Considering the tight linkage between SHANK3/SRRM2 and ASD pathogenesis, we utilized AF3 to analyze phosphorylation-triggered potential structural changes across different modification sites of the two proteins.

**Figure 7:**
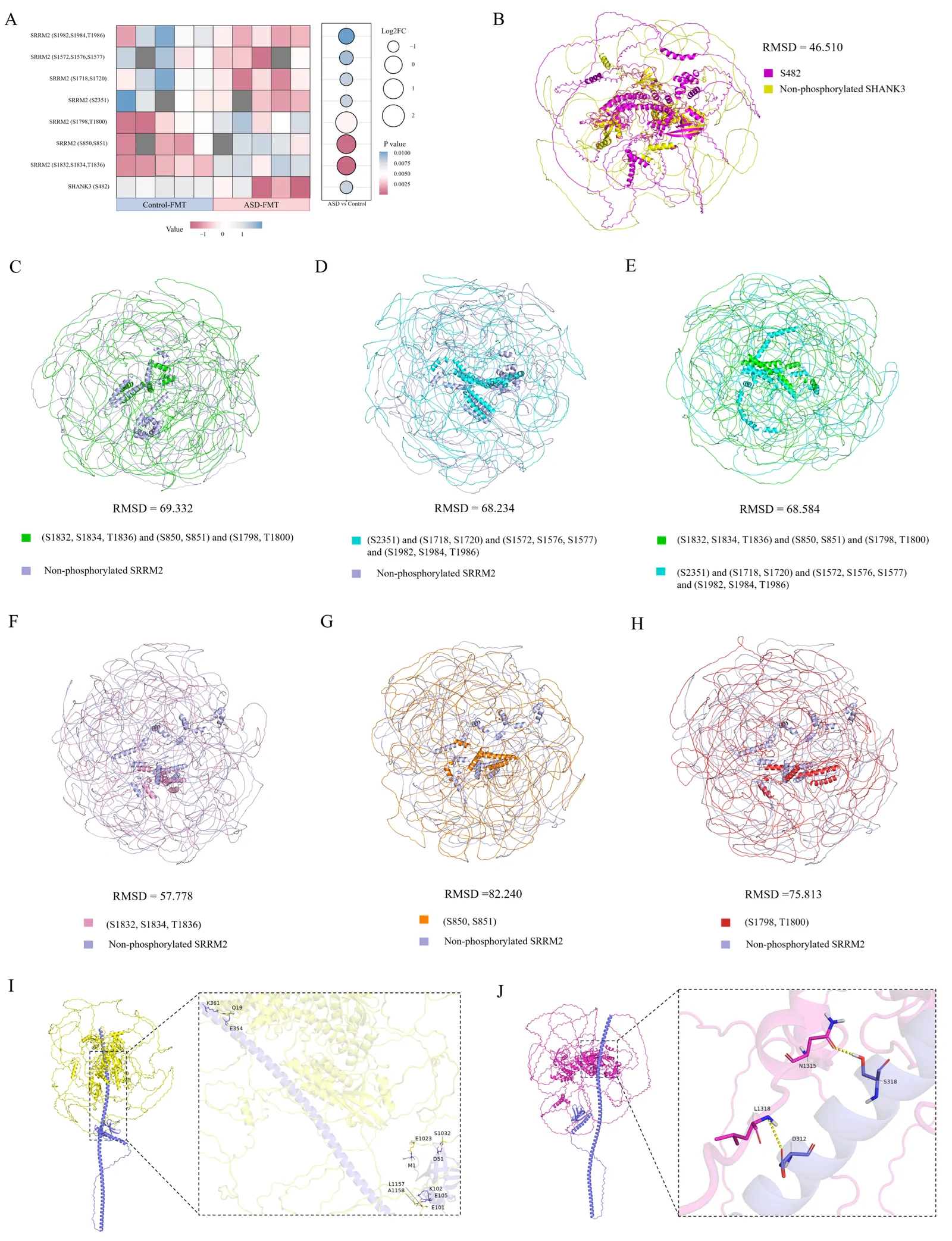
In silico structural modeling of SRRM2/SHANK3 and molecular docking simulations of the SHANK3–Homer1 complex under phosphorylated and non-phosphorylated states in the ASD-FMT and Control-FMT groups. (**A**) Heatmap illustrating the differential phosphorylation sites of target proteins between the ASD-FMT and Control-FMT groups. (**B**) AlphaFold 3 (AF3)-based prediction of protein conformational variability induced by hyperphosphorylation at the S482 site of SHANK3. (**C**–**H**) AF3-mediated structural prediction of SRRM2 conformational variations at distinct phosphorylated and non-phosphorylated states. (**I**,**J**) HADDOCK molecular docking simulations of binding interactions between the Homer1 ligand and two SHANK3 isoforms: (**I**) non-phosphorylated SHANK3; (**J**) phosphorylated SHANK3 at S482. In the structural diagrams, non-phosphorylated proteins are labeled in yellow, and S482-phosphorylated proteins are labeled in purple. Homer1 is shown in pink. Key amino acid residues at the binding interface are presented as sticks with their respective colors, and hydrogen bonds are indicated by yellow dashed lines.

Phosphoproteomic analysis revealed that gut microbiota derived from children with ASD induce hyperphosphorylation of SHANK3 at the S482 site (Figure 7A). Moreover, gut microbiota derived from ASD upregulated SRRM2 phosphorylation at S1832, S1834, T1836, S850, S851, S1798, and T1800, while downregulated at S2351, S1718, S1720, S1572, S1576, S1577, S1982, S1984, and T1986 (Figure 7A). We simulated and compared phosphorylation-induced conformational changes in SRRM2 and SHANK3 proteins using root mean square deviation (RMSD) values predicted by AF3. The phosphorylated SRRM2 and SHANK3 proteins were predicted to exhibit prominent in silico structural changes compared with their non-phosphorylated states, as indicated by RMSD values above 60 Å and 46.510 Å, respectively (Figure 7B–E). Furthermore, individual and combined phosphorylation sites may modulate in silico predicted structural models of SRRM2, with RMSD values exceeding 50 Å (Figure 7F–H). These in silico results indicated that aberrant phosphorylation of SHANK3 and SRRM2 proteins may give rise to conformational changes.

SHANK3 encodes a scaffolding protein critical for synaptic function and binds to the synaptic adaptor Homer to assemble complexes regulating synaptic signaling and plasticity [51,53,54]. Aberrant function of SHANK3 and its protein interactors drives synaptic dysfunction, a defining phenotypic hallmark of ASD [51,53,55]. Accordingly, we employed HADDOCK (High Ambiguity Driven DOCKing)-based molecular docking to predict whether hyperphosphorylation of the S482 site alters the binding of SHANK3 to Homer1. The results indicated that the binding free energy of non-phosphorylated SHANK3 bound to Homer1 was −5.4 kcal/mol (Figure 7I), whereas that of overphosphorylated SHANK3 at S482 bound to Homer1 reached −1.8 kcal/mol (Figure 7J). Our findings from molecular docking simulations first revealed that hyperphosphorylation of SHANK3 at S482 may reduce its binding ability with Homer1 (ΔΔG = −3.6 kcal/mol). Taken together, gut microbiota derived from children with ASD may promote S482 hyperphosphorylation of SHANK3; this abnormal post-translational modification could potentially remodel SHANK3 structural conformation, possibly attenuating its binding to Homer1.

### 3.8. The Gut Microbiota from Children with ASD Induced the Upregulation of FNDC3A

**Figure 8:**
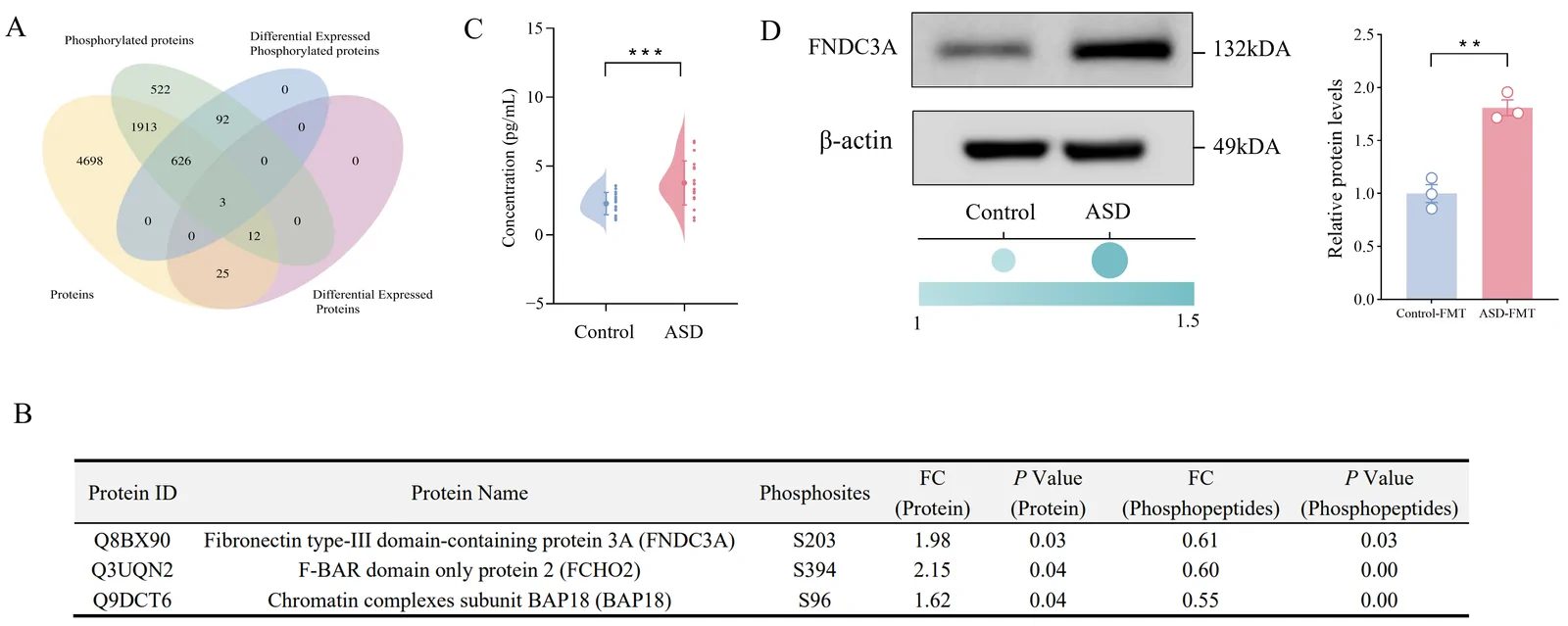
Integrated proteomic and phosphoproteomic analyses and cross-validation in mouse tissues and human samples. (**A**) Venn diagram showing the overlaps among total identified proteins, differentially expressed proteins, phosphorylated proteins, and differentially phosphorylated proteins. (**B**) Detailed differential expression information of the three overlapping proteins, including FNDC3A, FCHO2, and BAP18. (**C**) Comparative analysis of plasma FNDC3A expression levels in children with ASD and typically developing control children (*n* = 20 subjects per group). (**D**) Representative Western blot images and quantitative analysis of FNDC3A expression in hippocampal tissues from ASD-FMT and Control-FMT mice. Data represent n=3 biological replicates per group, with each sample analyzed in technical triplicate across independent experiments. Data are presented as means ± SEM. Statistical significance: ** *p* < 0.01, *** *p* < 0.001.

Furthermore, we integrated the proteomic and phosphoproteomic analyses to identify three overlapping proteins, as shown in the Venn diagram (Figure 8A,B). The *p*-values and FC of the three overlapping proteins, along with their respective phosphorylation sites, are listed in Figure 8B. We noted that FNDC3A exhibited higher expression, and its phosphorylation at the S203 site was significantly decreased in the ASD-FMT group compared with the Control-FMT group. The ELISA assay further confirmed that FNDC3A levels were also markedly higher in plasma samples of children with ASD compared with healthy control children (Figure 8C). We also performed Western blot analysis to verify that FNDC3A expression was significantly increased in the hippocampus of the ASD-FMT group (Figure 8D). Prior studies have documented copy number duplications and single-nucleotide polymorphisms (SNPs) of FNDC3A in children with ASD, suggesting FNDC3A mutations may affect susceptibility to this polygenic disorder [40,56]. Our study further observed that gut microbiota from children with ASD can elevate FNDC3A expression, which may serve as a potential pathogenic factor for autism.

## 4. Discussion

Accumulating evidence from human studies and animal research underscored the profound impact of gut microbiota dysbiosis on host brain function and social behaviors, demonstrating the important role of the gut–brain axis as a bidirectional communication system linking the gut and the brain. In this study, we employed an integrated approach combining in silico modeling with experimental validation to investigate how gut microbiota derived from children with ASD contribute to disease pathogenesis.

Mice colonized with gut microbiota from children with ASD exhibited several ASD-like behavioral abnormalities, including marked social novelty deficits, hypoactivity, anxiety-related behavior, and repetitive behaviors. These findings indicate that gut microbiota derived from children with ASD may drive both several core autism-related manifestations and some comorbid traits, such as reduced locomotor activity and elevated anxiety. Reduced motor activity and elevated anxiety are highly prevalent comorbidities among children with ASD and can independently restrict their active social interactions [57,58,59]. Our observations align with established animal models of ASD, including prenatal valproic acid (VPA) exposure and MECP2 mutant model [9,60,61,62].

At the cellular level, the gut microbiota derived from children with ASD triggered aberrant activation of hippocampal microglia and astrocytes. We also observed an elevated number of c-Fos-positive cells, indicating excessive neuronal activation within the hippocampus of ASD-FMT recipient mice. We next investigated neurogenesis in the dentate gyrus and observed a reduced immunoreactivity of doublecortin (DCX), a canonical marker of immature neurons, in the ASD-FMT group. Collectively, all the above results indicate that gut microbiota from children with ASD triggers aberrant activation of microglia, astrocytes and neurons, as well as disrupting neurogenesis, with these pathological alterations jointly contributing to neurodevelopmental deficits. Especially in the ASD pathophysiological state, reactive astrocytes and microglia lead to disturbances in synaptic activity, as well as neuronal signaling, which is referred to as synaptic dysfunction [63]. Such pathological alterations facilitate aberrant microglial pruning and astrocyte-induced synaptic dysfunction, followed by the development of the symptoms of ASD-like [63,64,65].

To unravel the molecular cascades underlying these cellular phenotypes, we performed hippocampal proteomic and phosphoproteomic profiling. In proteomics analysis, the differentially expressed proteins were selected by calculating the fold changes for the two groups, with ratios >1.5 or <0.67 defined as upregulated or downregulated, respectively. Only 29 proteins were differentially expressed between the ASD-FMT and Control-FMT groups, which were mainly involved in B cell differentiation and phosphorylation-dependent protein-binding pathway in GO and KEGG analyses. However, the phosphoproteomic results showed that up to 479 phosphorylated peptides were significantly changed, which mostly participate in nervous system development, synapse, postsynaptic density, postsynapse and presynapse, structural constituent of postsynapse, etc.

We then proceeded to verify that dendritic spine density of hippocampal neurons as well as presynaptic protein SYP and postsynaptic protein PSD-95 expression significantly decreased in the ASD-FMT group. SYP and PSD95 are canonical markers for presynaptic and postsynaptic elements, respectively, and also represent indicators of synaptic plasticity associated synapse formation and reconstruction [66]. Reduced expression of these two proteins is indicative of synaptic deficiency [67,68]. Western blotting demonstrated downregulated hippocampal SYP and/or PSD95 expression in children with ASD and valproic acid (VPA)-exposed rat model, corroborating the findings observed in our ASD-FMT recipient mice [69,70]. Gut microbiota from children with ASD may contribute to synaptic impairment through aberrantly phosphorylated proteins involved in synapse-related pathways.

These results suggested that gut microbiota derived from children with ASD may lead to hippocampal synaptic dysfunction through abnormal phosphorylation, which may be one of the causative factors and contribute to ASD progression. Nevertheless, there are also only a few altered synapse-related pathways, such as reduced postsynaptic structural constituents and dysregulated MECP2 expression and activity, identified merely in the GSEA based on proteomic datasets. In addition, our phosphoproteomic study targeting hippocampal regions identified key signaling pathways, such as MAPK signaling, Rap1 signaling, calcium signaling and cAMP signaling pathways, which can regulate the blood–brain barrier as shown in previous accumulated research [46,47,71,72,73]. Furthermore, we used Western blot analysis to verify that impairment of blood–brain barrier integrity existed, followed by ASD-FMT, which may occur through abnormal protein phosphorylation. However, the proteomic data and analysis did not, or rarely, reveal any above similar signals. Therefore, molecular signals related to the ASD pathogenic mechanism detected by phosphoproteomic analysis were more relevant to changes by the gut microbiota from children with ASD than those detected by proteomic analysis alone.

To evaluate whether aberrant phosphorylation drives conformational and functional alterations in key neurodevelopmental proteins, we conducted computational structural modeling using AlphaFold3 (AF3) and HADDOCK docking simulations on SHANK3 and SRRM2. Recent transformative developments in deep learning, best exemplified by AlphaFold, have upended conventional protein structure prediction workflows, delivering prediction accuracies rivaling those derived from experimental structural analyses [17,50]. SHANK3 is known to play critical roles in synaptic structure and functions [53,74] Shank3 is a well-established autism risk gene, and detailed mechanistic studies provide insights into ASD pathogenesis [75,76]. SHANK3 serves as a scaffolding protein within the postsynaptic density (PSD) of dendritic spines, which are actin-rich protrusions from dendrites [77]. The copy-number variants, especially deletion or haploinsufficiency, in Shank3 gene were studied more prevalently in the field of autistic pathogenesis in human and animal models [14,75]. However, phosphorylation-induced structural alterations of the SHANK3 protein and the underlying molecular mechanisms remain understudied. In this study, we simulated phosphorylation-triggered hypothetical structural changes in SHANK3 protein using AF3 and molecular docking to predict the binding of SHANK3 to the Homer1 using HADDOCK. Our results indicated that the gut microbiota derived from children with ASD induces hyperphosphorylation of SHANK3 at the S482 site, which may lead to potential structural changes compared with its non-phosphorylated state (RMSD = 46.510 Å) and attenuate its binding to Homer1 (ΔΔG = −3.6 kcal/mol). SHANK3 mutations reduce the expression of postsynaptic density (PSD) proteins in neurons, including guanylate kinase-associated protein (GKAP), HOMER1b/C, the AMPA receptor subunit GluA1 and the NMDA receptor subunit NR2A [10,14,78]. Our results indicate prominent reductions in PSD95 abundance, which may be ascribed to aberrant SHANK3 phosphorylation that triggers potential structural synaptic malformations and compromises putative protein docking interactions. Integrating the previous outcomes with our own results highlights that SHANK3 serve as vital regulators of hippocampal functional homeostasis [10,14,53,75]. Furthermore, we simulated abnormal phosphorylation-triggered structural changes in SRRM2 protein. Previous studies have shown that SRRM2 variants are closely associated with neurodevelopmental disorders and autism [79,80,81]. In our study, we found abnormal individual and combined phosphorylation sites could potentially modulate SRRM2 structural alterations. Therefore, gut microbiota dysbiosis raises the possibility of inducing aberrantly phosphorylated proteins and consequent structural modifications, a process that may serve as a key functional component in the molecular mechanisms of autism pathogenesis.

We integrated proteomic and phosphoproteomic profiling and identified three shared proteins, namely FNDC3A, FCHO2, and BAP18. We focused on FNDC3A, as FNDC3A genetic polymorphisms and locus duplications have been previously linked to ASD susceptibility [40,56]. Our integrated proteomic and phosphoproteomic profiling results show that FNDC3A upregulated levels, and phosphorylation of FNDC3A at S203 was substantially attenuated in the hippocampal tissue of recipient mice subjected to FMT using fecal samples from children with ASD. Western blot analysis validated elevated hippocampal FNDC3A expression in recipient mice, and ELISA assays confirmed elevated circulating FNDC3A levels in plasma from children with ASD. These results highlight FNDC3A as a promising susceptibility candidate modulated by the gut microbiota.

This study has several limitations that warrant acknowledgment. Firstly, structural models generated by AF3 and HADDOCK represent predictive working hypotheses. High global RMSD values can partly stem from baseline conformational shifts in intrinsically disordered regions. Future empirical studies utilizing NMR, cryo-EM, small-angle X-ray scattering (SAXS), biophysical binding assays, and site-directed mutagenesis (e.g., S482 phosphomutants) are essential to empirically validate the predicted structural rearrangements and protein–protein interactions. While FNDC3A upregulation was established across mouse brain tissues and human plasma, loss- and gain-of-function studies (e.g., knockdown/overexpression) are needed to delineate its precise pathogenic role. Similarly, direct in vivo functional assays (such as Evans Blue extravasation or TRITC-dextran tracer permeability) are required to functionally evaluate BBB integrity. Lastly, given the high clinical heterogeneity of ASD, the microbiota alterations identified in our cohort are cohort-specific and cannot be generalized to all individuals with ASD. Variations in age, comorbidities, lifestyle and medication status across different populations may shape distinct microbial profiles. Thus, our findings do not define a universal ASD microbiota signature and should not be interpreted as absolute, generalized conclusions. Furthermore, using pooled human fecal samples for FMT can allow individual samples with extreme dysbiosis to disproportionately skew the overall donor profile.

## 5. Conclusions

Collectively, gut microbiota derived from children with ASD may drive pathogenesis through multifaceted mechanisms. These include the induction of ASD-like behavioral phenotypes, excessive activation of glial cells and neurons, impaired neurogenesis, downregulation of synapse-related proteins and signaling pathways, and compromised expression of key BBB tight-junction proteins. Notably, phosphoproteomic profiling revealed that molecular signaling pathways, particularly the recurrent synapse-associated pathways identified via KEGG and GO enrichment analyses, correlated more strongly with gut microbiota alterations in autistic children than signatures uncovered by conventional proteomics alone.

Furthermore, we established an integrative research framework. First, proteomic and phosphoproteomic profiling was performed to identify candidate proteins carrying aberrant phosphorylation sites may link to gut microbiota-driven ASD pathogenesis. We then integrated in silico structural prediction and molecular docking simulations, followed by comprehensive wet-lab assays to validate synaptic pathways, target proteins, key BBB tight junction proteins, and so on. Ultimately, while monogenic mutational disruptions (e.g., duplications and deletions) leading to aberrant gene expression are well-established in ASD, our findings suggest that gut microbiota-driven post-translational modifications (e.g., protein phosphorylation) and subsequent structural remodeling may also be a key contributor to the initiation of ASD. Future mechanistic investigations into ASD cannot overlook how environmental regulatory factors, particularly gut microbiota, modulate protein post-translational modifications to promote the ASD pathogenesis.

## Data Availability

The original contributions presented in this study are included in the article/supplementary material. Further inquiries can be directed to the corresponding author.

## Supplementary Materials

The following supporting information can be downloaded at: https://www.mdpi.com/article/10.3390/microorganisms14091925/s1; Supplementary Table S1: Per-sample sequencing and quality control summary. Supplementary Table S2: Relative abundances of phylum-level microbiota between Control-pre ABX and Control-post ABX. Supplementary Table S3: Relative abundances of phylum-level microbiota between ASD-pre ABX and ASD-post ABX. Supplementary Table S4: HADDOCK 2.4 parameters setting.
