# Supplementary material for: Gut Microbiota-Elicited Aberrant Phosphorylation Induces Protein Structural Anomalies: A Non-Negligible Pathogenic Driver of Autism Spectrum Disorder
Source: Microorganisms. 2026 Sep 1;14(9):1925. doi: 10.3390/microorganisms14091925 (PMC13608970; doi:10.3390/microorganisms14091925)
Supplement: Supplementary file 1 [file microorganisms-14-01925-s001.zip › microorganisms-4450760-supplementary.pdf]

Supplementary Table S1. Per-sample sequencing and quality control summary.

Sample	Raw Reads	Raw Bases	Valid Tags	Valid Bases	Valid%	Q20%	Q30%	GC%
A101	89740	53.84M	82297	34.50M	91.71	99.67	98.95	53.41
A102	86137	51.68M	78510	32.81M	91.15	99.68	99.00	53.21
A103	81340	48.80M	76033	31.87M	93.48	99.60	98.73	53.43
A104	85917	51.55M	80756	33.76M	93.99	99.40	98.08	53.74
A105	58930	35.36M	55467	23.03M	94.12	99.18	97.52	53.94
A106	85923	51.55M	80170	33.41M	93.30	99.85	99.56	53.09
A107	86617	51.97M	82829	34.46M	95.63	99.82	99.46	53.39
A108	77350	46.41M	69781	29.20M	90.21	99.56	98.63	53.25
A109	71017	42.61M	65527	27.24M	92.27	99.72	99.08	53.09
A110	86505	51.90M	79906	33.35M	92.37	99.66	98.92	53.96
A111	84970	50.98M	78996	32.71M	92.97	99.67	98.96	53.64
A112	67803	40.68M	62292	26.02M	91.87	99.55	98.58	53.17
A113	76401	45.84M	71704	29.84M	93.85	99.44	98.25	53.86
A114	80857	48.51M	74748	31.31M	92.44	99.68	98.97	54.21
A115	70681	42.41M	66425	27.56M	93.98	99.57	98.64	53.69
A201	85367	51.22M	83427	35.60M	97.73	99.84	99.52	53.85
A202	84064	50.44M	82407	35.17M	98.03	99.60	98.82	53.85
A203	87974	52.78M	85775	36.61M	97.50	99.51	98.47	53.84
A204	81536	48.92M	79737	34.03M	97.79	99.79	99.36	53.85
A205	81489	48.89M	77551	32.01M	95.17	99.79	99.36	52.91
A206	87345	52.41M	82208	34.49M	94.12	99.79	99.36	53.47
A207	65453	39.27M	62704	26.62M	95.80	99.60	98.70	53.85
A208	88560	53.14M	83130	34.90M	93.87	99.52	98.45	51.97
A209	83957	50.37M	75103	31.04M	89.45	99.67	98.91	53.68
A210	75512	45.31M	67929	27.95M	89.96	99.59	98.70	53.90
A211	86371	51.82M	74992	30.90M	86.83	99.70	99.01	53.58
A212	83412	50.05M	74767	30.89M	89.64	99.66	98.88	53.73
A213	80684	48.41M	74605	30.87M	92.47	99.73	99.18	53.72
A214	81116	48.67M	74949	30.46M	92.40	99.63	98.88	52.01
A215	85638	51.38M	80123	33.46M	93.56	99.65	98.97	53.21
A301	83320	49.99M	73900	30.73M	88.69	99.41	98.15	50.33
A302	84196	50.52M	76141	31.51M	90.43	98.54	96.03	50.99
A303	76004	45.60M	67357	27.98M	88.62	99.45	98.27	50.96
A304	71737	43.04M	63704	26.48M	88.80	98.98	96.72	50.54
A305	87246	52.35M	77950	32.37M	89.35	99.66	98.83	50.39
A306	78272	46.96M	69151	28.70M	88.35	99.65	98.86	50.73
A307	77565	46.54M	69407	28.69M	89.48	99.18	97.33	51.56
A308	72566	43.54M	61920	25.81M	85.33	99.37	97.99	50.20
A309	83886	50.33M	73748	30.71M	87.91	99.65	98.87	49.98

Sample	Raw Reads	Raw Bases	Valid Tags	Valid Bases	Valid%	Q20%	Q30%	GC%
A310	87212	52.33M	75553	31.45M	86.63	99.34	97.86	50.11
A311	84043	50.43M	74905	31.12M	89.13	99.51	98.44	50.80
A312	82065	49.24M	72335	29.98M	88.14	99.60	98.72	51.10
A313	84214	50.53M	70371	29.22M	83.56	99.44	98.23	50.57
A314	87463	52.48M	76004	31.73M	86.90	99.56	98.62	50.15
A315	86750	52.05M	77599	32.21M	89.45	99.63	98.81	50.73
B101	81735	49.04M	76925	31.86M	94.12	99.53	98.55	54.09
B102	75766	45.46M	70642	29.48M	93.24	99.42	98.13	54.34
B103	87385	52.43M	80225	33.33M	91.81	99.48	98.39	53.16
B104	87232	52.34M	81076	34.10M	92.94	99.64	98.84	52.67
B105	85134	51.08M	79617	33.30M	93.52	99.26	97.66	53.87
B106	86328	51.80M	81965	34.09M	94.95	99.83	99.51	54.42
B107	89417	53.65M	81723	34.09M	91.40	99.86	99.60	52.68
B108	84153	50.49M	79370	33.13M	94.32	99.72	99.15	53.35
B109	59551	35.73M	53986	22.59M	90.66	99.46	98.29	53.59
B110	66357	39.81M	62114	25.97M	93.61	99.67	98.96	54.06
B111	73107	43.86M	69911	29.27M	95.63	99.36	98.05	54.81
B112	72189	43.31M	67690	28.35M	93.77	98.84	96.54	53.64
B113	85751	51.45M	80914	33.64M	94.36	99.56	98.62	54.08
B114	87429	52.46M	80675	33.59M	92.27	99.65	98.89	53.58
B115	89787	53.87M	80848	33.83M	90.04	99.67	98.96	53.55
B201	87807	52.68M	79919	32.85M	91.02	99.81	99.42	54.12
B202	83777	50.27M	69416	28.38M	82.86	99.74	99.16	53.35
B203	81800	49.08M	79204	33.79M	96.83	99.47	98.33	53.86
B204	83027	49.82M	80728	34.44M	97.23	99.75	99.22	53.86
B205	60366	36.22M	58726	25.04M	97.28	99.88	99.63	53.84
B206	82570	49.54M	80736	34.44M	97.78	99.57	98.71	53.84
B207	80744	48.45M	78616	33.55M	97.36	99.38	98.01	53.85
B208	86208	51.72M	80246	32.72M	93.08	99.66	98.95	52.86
B209	59746	35.85M	55025	21.58M	92.10	99.64	98.87	51.56
B210	86750	52.05M	82486	33.86M	95.08	99.60	98.77	53.62
B211	55795	33.48M	52963	22.60M	94.92	99.63	98.86	52.71
B212	82397	49.44M	79592	33.97M	96.60	99.55	98.56	52.70
B213	61425	36.85M	58550	24.53M	95.32	99.30	97.84	53.54
B214	63786	38.27M	61248	25.40M	96.02	99.48	98.35	53.84
B215	52608	31.56M	50345	20.15M	95.70	98.98	96.98	51.96
B301	78691	47.21M	62892	26.07M	79.92	99.66	98.90	51.38
B302	85060	51.04M	69989	28.99M	82.28	99.60	98.72	50.71
B303	82640	49.58M	69329	28.70M	83.89	98.76	96.54	50.81

Sample	Raw Reads	Raw Bases	Valid Tags	Valid Bases	Valid%	Q20%	Q30%	GC%
B304	84502	50.70M	66334	27.57M	78.50	99.64	98.86	51.06
B305	66239	39.74M	51627	21.39M	77.94	99.66	98.93	50.91
B306	87777	52.67M	69879	29.07M	79.61	99.50	98.42	50.60
B307	88540	53.12M	70133	29.22M	79.21	99.36	97.99	50.85
B308	72433	43.46M	53606	22.24M	74.01	99.60	98.74	50.73
B309	84678	50.81M	68388	28.32M	80.76	99.60	98.69	51.23
B310	80200	48.12M	63823	26.49M	79.58	99.65	98.93	50.44
B311	66615	39.97M	51418	21.33M	77.19	99.54	98.55	50.80
B312	83131	49.88M	62923	26.14M	75.69	99.62	98.78	50.59
B313	84245	50.55M	66709	27.60M	79.18	99.56	98.61	51.33
B314	84759	50.86M	64379	26.77M	75.96	99.30	97.80	50.35
B315	77938	46.76M	62628	25.96M	80.36	99.08	97.25	50.70

Supplementary Table S2. Relative abundances of phylum-level microbiota between Control-pre ABX and Control-post ABX.

Object	Control-pre ABX	Control-post ABX
Others	0.036668378	0.24202743
p__Myxococcota	0.000333349	0.085009634
p__Chloroflexota	0	0.086009748
p__Cyanobacteriota	0.011000513	0.361040918
p__Thermodesulfobacteriota	0.265679065	0.143682951
p__Patescibacteria	0.891041582	0.437382903
p__Actinomycetota	0.231010781	3.536734163
p__Verrucomicrobiota	8.14171328	0.674409766
p__Bacillota	22.50338349	17.06426728
p__Bacteroidota	65.81640477	3.140689278
p__Pseudomonadota	2.102764796	74.22874592

Supplementary Table S3. Relative abundances of phylum-level microbiota between ASD-pre ABX and ASD-post ABX.

Object	ASD-pre ABX	ASD-post ABX
Others	0.019333527	0.21300071
p__Deferribacterota	0.088667553	0.014666716
p__Myxococcota	0	0.121333738
p__Cyanobacteriota	0.006333397	0.319667732
p__Thermodesulfobacteriota	0.261335947	0.154667182
p__Patescibacteria	1.300013	0.974336581
p__Actinomycetota	0.281669483	2.159340531
p__Verrucomicrobiota	12.97979646	1.79100597
p__Bacillota	18.50085168	20.13206711
p__Pseudomonadota	2.61802618	66.32888776
p__Bacteroidota	63.94397277	7.79102597

Supplementary Table S4. HADDOCK 2.4 parameters setting

Energy terms	it0 (rigid-body)	it1 (semi-flexible)	itw (water refinement)
van der Waals (vdw)	0.01	1.0	1.0
Electrostatics (elec)	1.0	1.0	0.2
Desolvation (desolv)	1.0	1.0	1.0
Ambiguous interaction restraints (AIR)	0.01	0.1	1.0
Buried surface area (BSA)	-0.01	-0.01	0.0
Radius of gyration (Rg)	0.1	1.0	1.0
Symmetry restraints (sym)	0.1	0.1	0.1
